# Toward a cohesive understanding of ecological complexity

**DOI:** 10.1126/sciadv.abq4207

**Published:** 2023-06-21

**Authors:** Federico Riva, Caio Graco-Roza, Gergana N. Daskalova, Emma J. Hudgins, Jayme M. M. Lewthwaite, Erica A. Newman, Masahiro Ryo, Stefano Mammola

**Affiliations:** ^1^ Geomatics and Landscape Ecology Laboratory, Department of Biology, Carleton University, 1125 Colonel By Dr, Ottawa, Ontario K1S 5B6, Canada.; ^2^ Insectarium, Montreal Space for Life, 4581 Sherbrooke St E, Montreal, Quebec H1X 2B2, Canada.; ^3^ Spatial Ecology Group, Department of Ecology and Evolution, Université de Lausanne, Lausanne, Switzerland.; ^4^ Aquatic Community Ecology Group, Department of Geosciences and Geography, University of Helsinki, Gustaf Hällströmin katu 2, 00560 Helsinki, Finland.; ^5^ Laboratory of Ecology and Physiology of Phytoplankton, Department of Plant Biology, State University of Rio de Janeiro, Rua São Francisco Xavier 524, PHLC, Sala 511a, 20550-900 Rio de Janeiro, Brazil.; ^6^ Biodiversity and Ecology Group, International Institute for Applied Systems Analysis, Laxenburg, Austria.; ^7^ Marine and Environmental Biology, University of Southern California, 3616 Trousdale Pkwy, Los Angeles, CA 90089-0371, USA.; ^8^ Department of Integrative Biology, University of Texas at Austin, Austin, TX 78712, USA.; ^9^ Leibniz Centre for Agricultural Landscape Research (ZALF), Eberswalder Str. 84, 15374 Muencheberg, Germany.; ^10^ Environment and Natural Sciences, Brandenburg University of Technology Cottbus-Senftenberg, 03046 Cottbus, Germany.; ^11^ Laboratory for Integrative Biodiversity Research (LIBRe), Finnish Museum of Natural History (LUOMUS), University of Helsinki, Pohjoinen Rautatiekatu 13, Helsinki 00100, Finland.; ^12^ Molecular Ecology Group (MEG), Water Research Institute (IRSA), National Research Council (CNR), Corso Tonolli, 50, Pallanza 28922, Italy.; ^13^ National Biodiversity Future Center, Palermo, Italy.

## Abstract

Ecological systems are quintessentially complex systems. Understanding and being able to predict phenomena typical of complex systems is, therefore, critical to progress in ecology and conservation amidst escalating global environmental change. However, myriad definitions of complexity and excessive reliance on conventional scientific approaches hamper conceptual advances and synthesis. Ecological complexity may be better understood by following the solid theoretical basis of complex system science (CSS). We review features of ecological systems described within CSS and conduct bibliometric and text mining analyses to characterize articles that refer to ecological complexity. Our analyses demonstrate that the study of complexity in ecology is a highly heterogeneous, global endeavor that is only weakly related to CSS. Current research trends are typically organized around basic theory, scaling, and macroecology. We leverage our review and the generalities identified in our analyses to suggest a more coherent and cohesive way forward in the study of complexity in ecology.

## Teaser

Progress in ecology and conservation requires weaving the study of ecological complexity with complex system science.

## INTRODUCTION

Understanding nature’s complexity is at the core of science (*1*–*6*). In ecology and conservation, studying complexity has led to both the development of theories (*2*, *7*–*11*) and considerations in policies and plans for environmental management (*12*–*16*). Understanding complexity is also becoming increasingly important in the face of accelerating global environmental change, because ecological systems exposed to multiple stressors often display phenomena typical of complex systems (*14*, *15*, *17*–*19*). Advancements in the study of complexity are therefore crucial, which has been recognized in the 2021 Nobel Prize in Physics, awarded to Parisi, Manabe, and Hasselmann for their “groundbreaking contributions to our understanding of complex systems” (*20*).

Complexity sciences belong in a central role in ecology and conservation because ecological systems are quintessentially complex systems (*3*, *18*, *21*). Understanding the complexity of ecological systems may, therefore, be key for addressing ongoing environmental crises (*19*, *22*, *23*). For instance, the risk that climate change will result in abrupt shifts in Earth’s climate is considerable, and this awareness is critical to informing climate policy aimed at preventing catastrophic scenarios (*15*, *24*). Climate change, in turn, affects ecosystems and food webs that are already degraded and altered from millennia of human activities worldwide (*25*, *26*). Recognition of the potential for a planetary systemic failure due to climate-biodiversity feedback is urgent and increasingly recognized (*14*, *19*, *22*, *27*). Nevertheless, untangling complex dynamics of ecological systems is hindered by the fact that such systems are rarely studied as complex systems per se. Most research in the environmental sciences continues to follow traditional reductionist approaches (*28*) instead of applying preexisting developments in complex system science (CSS).

CSS emerged in the 20th century from a confluence of disciplines that independently attempted to bypass the limitation of conventional, reductionist approaches in the study of complex systems (*6*, *29*, *30*). CSS is tied to more traditional studies on complexity, which attempts to understand the fundamental, governing laws of complexity, but it distinguishes itself as an independent, quantitative field that attempts to identify and explain phenomena across complex systems of different types, including the biological, social, and technological. Despite the historical epistemological and ontological difficulties in defining complexity (*3*, *31*–*33*), researchers in CSS have reached a consensus on what characterizes complex systems (*4*–*6*, *18*, *21*, *34*, *35*) (Table 1). Grounding the study of ecological complexity in CSS therefore has the potential to facilitate coordinated advancements in ecology and the study of complexity. Here, we provide a synthesis to identify and forge links between these disciplines.

**Table 1.** Features typical of complex ecological systems. Features identified as typical of complex ecological systems through a critical review of the literature in complexity science. Note that search strings are presented as word stem (e.g., “self-orga”) to capture plurals and alternative forms and spellings (e.g., self-organization, self-organisation, self-organising, etc.). We introduce a short definition of each feature with some seminal references for brevity, but it does not necessarily exclude other definitions. See “What makes a system complex?” and “Identifying features typical of ecological complexity” sections in the text for more details.

Feature	Definition	Search string	Related concepts
Adaptation	The parts and/or a system change in response to changes in external or internal factors or states (*4*, *18*)	adapt	Evolution, niche, plasticity, phenological shifts
Aggregation	The parts that compose a system tend to organize into groups (*18*, *88*)	aggregat	Consortia, superstructures
Attractor	One of many states toward which a system tends to evolve (*71*, *111*)	attractor	Criticality, hysteresis, tipping points, stable states
Chaos	A type of dynamical system where small differences in the initial conditions result in great, deterministic differences among the potential states of that system (*60*, *74*)	chaos + chaotic	Sensitivity, phase space divergence
Diversity	The parts that compose a system are not equal (*5*, *86*)	diversit	Entropy, heterogeneity, information, variation
Dynamicity	The property of systems and parts change with time (*14*, *67*)	dynamic	Evolution, stasis, transformation
Emergence	The property of system characteristics that are not predictable based on the characteristics of their parts (*1*, *41*)	emergen	Collective intelligence, gestalt principles
Feedback	Processes in the system that increase or reduce the likelihood of the same process happening again (*67*, *103*)	feedback	Reinforcement, top-down causation
Flow	Exchange of material, energy, or information across the system (*7*, *21*)	flow	Information, linkages
Fractality	Self-similar regularities that repeat across scales (*71*, *114*)	fractal	Regularity, scale invariance
Hierarchy	The system exhibits properties at multiple organizational levels (*69*, *92*)	hierarch	Levels, nestedness, scales
Homeostasis	Self-regulating mechanisms maintaining a system functioning and persisting (*88*, *123*)	homeosta	Control, robustness
Interaction	The parts that compose a system affect each other (*13*, *41*)	interact	Competition, dependence, parasitism, mutualism, synergy
Memory	Previous states of the system influence present and future states (*72*, *90*)	memory + memories	Lagged responses, Markov processes
Modularity	The property whereby some parts of a system interact more strongly among themselves than with the rest (*8*, *18*)	modul	Cluster, connectivity, stability
Network	A representation of relationships (links) occurring between parts (nodes) in a system (*53*, *65*)	network	Food webs, feedbacks, nodes
Nonequilibrium	The state of a system that has not reach a steady state (*67*, *111*)	non-equilib + non equilib + nonequilib	Balance, disturbance, multiple stable states, instability
Nonlinearity	A property of systems where the change of the output is not proportional to the change of the input (*68*, *86*)	non-linear + non linear + nonlinear	Higher-order effects
Resilience	The capacity of a system to resist and recover from disturbance (*12*, *116*)	resilien	Brittleness, robustness, stability
Scaling	The property of system patterns to change with scale (e.g., spatial, temporal, or taxonomic) (*7*, *11*)	scal + scale-depend + scale depend	Discrete hierarchy, grain, levels
Self-organization	The emergence of global patterns, dynamics, or ordered structures from the local interaction among the components of a system (*5*, *88*)	self-orga + self orga + selforga	Evolution, emergence, multicellularity, pattern formation
Stability	The tendency of a system to return to its equilibrium state (*2*, *152*)	stabilit	Invasibility, persistence, resistance, robustness
Threshold	The context in which a small change in the conditions of a system results in large change in the system itself (*15*, *88*)	thresho	Criticality, tipping point

**Fig. 1.**
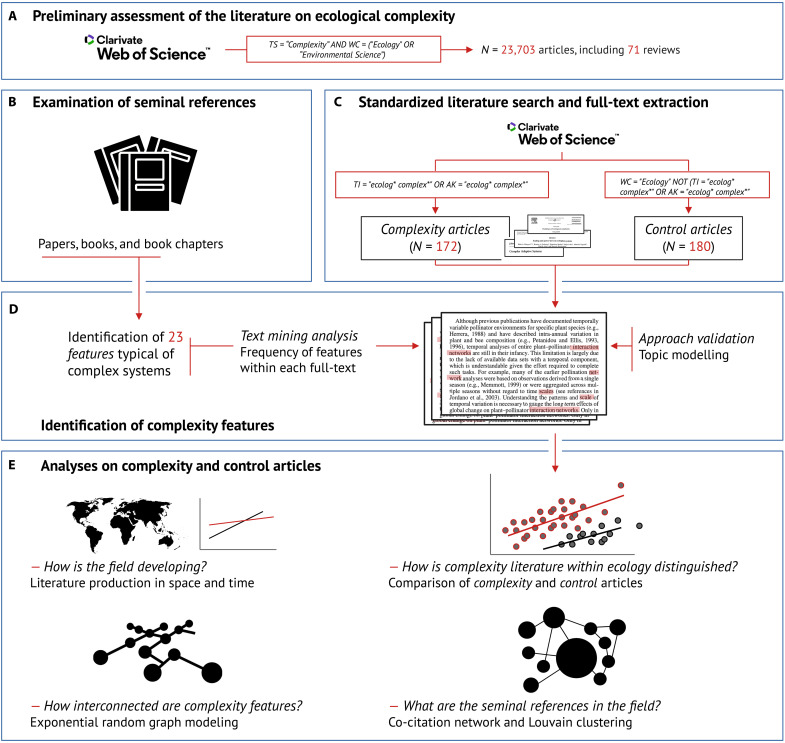
Analytical roadmap. Summary illustrating the stepwise process of data collection and analyses in this study. (**A**) Preliminary assessment of the literature done through a search on Web of Science. (**B**) Examination of the papers, books, and book chapters as well as (**C**) the standardized literature search and full-text extraction to search for the (**D**) 23 features identified on (B) in the full text of articles retrieved in (C). (**E**) Analyses on complexity and control articles in the search for generalities in the field of ecological complexity. TS, topic; WC, Web of Science categories; TI, title; AK, author keywords.

Coordination between ecology and CSS is needed because the study and invocation of ecological complexity continues to grow in the scientific literature, while there is also persistent imprecision in how ecologists use the term “complexity.” A search on the Web of Science for the word “Complexity” in the “Ecology” and “Environmental Sciences” categories matched 23,703 manuscripts published between 2000 and 2021 (search conducted on 14 July 2021; Fig. 1A). The 71 review articles captured by this search discuss a broad range of topics, from the evolutionary novelty of venoms (*36*) to the biogeochemistry of marine polysaccharides (*37*), but none directly addresses what ecological complexity is or how it emerges from lower levels (table S1). Instead, complexity is often used in a colloquial sense, implying that a study focuses on a system that is difficult to comprehend. In other words, “complex” is often mistaken by “complicated.” Based on the 71 review articles, the study of ecological complexity appears highly disorganized, with few common threads across an extensive body of literature. This lack of clarity will likely confound the communication of ideas, foster unnecessary debates, limit research progress, and hinder the translation of findings into practice (*38*). Given the importance of understanding natural systems in the face of global change, seeking common ground in how we study and define complexity is not merely a semantic problem but instead a pressing challenge for current science.

**Fig. 2.**
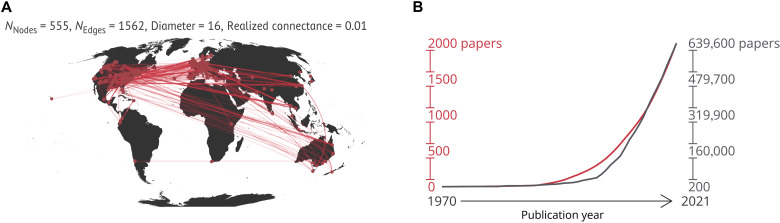
The study of ecological complexity in space and time. (**A**) Global network of collaborations including all authors from the articles that referred to “ecological complexity” in their title or keywords (*n* = 172). Points represent researchers’ affiliation addresses, and lines indicate collaboration between authors. (**B**) Cumulative production (from 1970 to 2021) between articles mentioning “complexity” in their titles and abstracts including all scientific fields (gray line) and, separately, for ecology and environmental sciences, as approximated by the search term “ecological complexity” (red line).

**Fig. 3.**
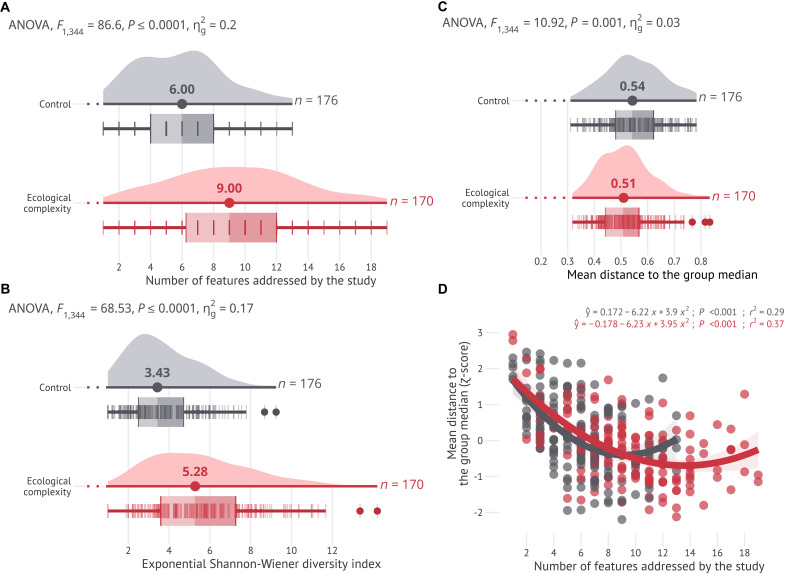
Comparison between control and complexity articles. Comparison between control (gray) and complexity (red) groups considering the features retrieved by the systematic mapping (listed in Table 1). The control group includes articles randomly selected from the ecological literature, and the complexity group includes articles explicitly referring to “ecological complexity” in their title or keywords. Note that six articles (control = 4, complexity = 2) did not include any of the features described in Table 1 and were excluded from the analysis. (**A**) The richness of features of each article and (**B**) the exponential of the Shannon entropy calculated on relative frequency of feature usage were significantly higher in the complexity articles. (**C**) Study uniqueness (i.e., the distance from each article to its group median) was smaller in complexity articles, indicating that these were typically more similar among themselves. (**D**) The relationship between study uniqueness and feature richness shows that articles mentioning fewer features were on average more distant from their group mean, suggesting that these features were rarely mentioned by other articles. In (A) to (C), the data distributions are depicted with a kernel density plot with a dot representing the median value, and a box-and-whisker plot with outliers representing the minimum, Q1, median, Q3, and maximum with the length of 1.5 × the interquartile range.

**Fig. 4.**
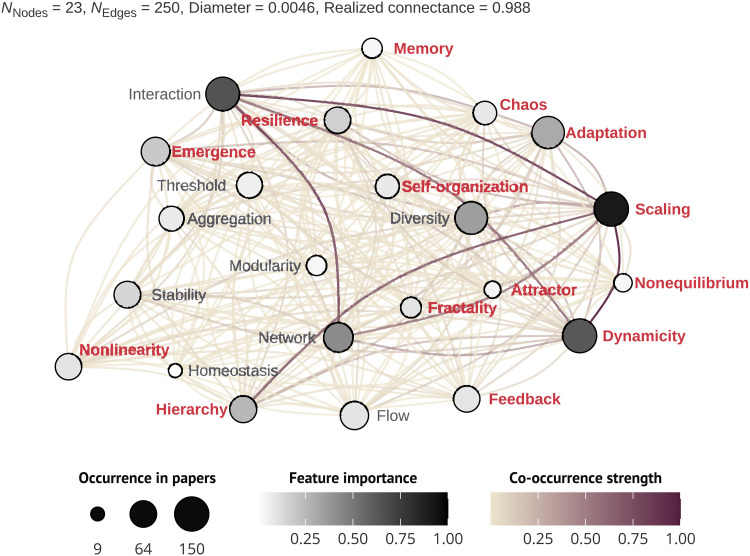
Connections among complexity features in ecology. This unipartite network shows the projection of a bipartite network linking complexity features (Table 1) based on their co-occurrence in the “complexity” group of articles. Features (nodes of the network) are shown, with more red color indicating that features are more significantly associated with the complexity articles based on indicator species analysis. Co-occurrence strength (edges) is represented by the sum of the edge weights of the adjacent edges of the node.

**Fig. 5.**
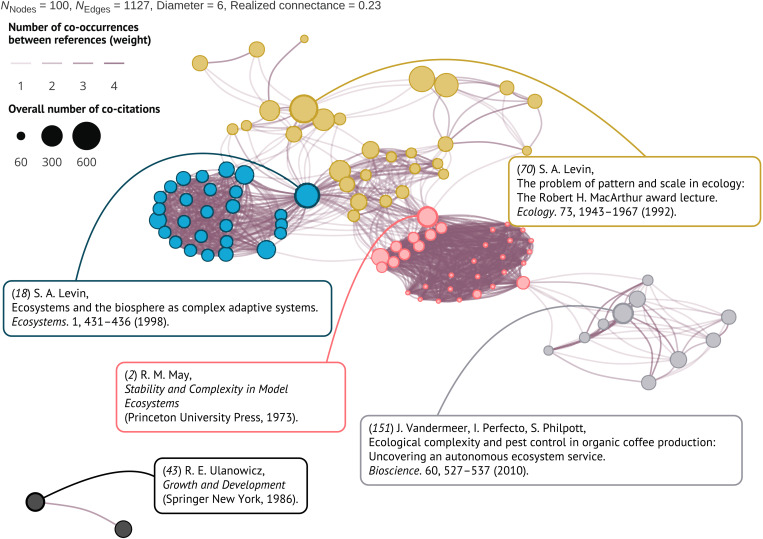
Seminal literature and the topic clusters in the ecological complexity literature. Weighted co-citation network for the top 100 co-cited articles in the complexity articles. The colors reflect co-citation clusters: foundational complexity theory [(*18*); in blue]; scaling, hierarchies, and cross-scale dynamics [(*70*); in gold]; and macroecological theory and large-scale systems [(*2*); in pink]. Two additional clusters [(*43*, *151**)*; in gray] count 10 or less articles and emerged from the use of “ecological complexity” in a more specific context [e.g., pest control in agriculture (*151*)].

To this end, here we combine the strengths of a critical review, text mining, and “science of science” (*39*) analyses (Fig. 1). We first assess how ecologists conceptualize complexity following a three-pronged approach: (i) we review CSS literature to identify a list of features typically attributed to complex ecological systems (Fig. 1B and Table 1); (ii) we assess the ecological literature to understand how these features relate to the study of “ecological complexity” (Fig. 1, C and D; results from the analyses illustrated in Figs. 2 to 5); and (iii) we leverage our critical review and generalities identified in our analysis to suggest a cohesive way forward in the study of complexity in ecology. This empirical approach allows us to face the longstanding challenges of understanding complexity: Instead of defining the study of complexity from first-principle reasoning, we quantitatively assess the literature to understand how ecologists have conceptualized complexity. Before addressing current practices and how to improve apparent confusion in the study of ecological complexity, we contextualize our work by providing a brief account of the history of CSS and by describing the philosophy underlying CSS within the broader study of complexity.

## A BRIEF HISTORY OF COMPLEX SYSTEM SCIENCE (CSS)

Understanding the history of CSS helps to appreciate why progress in the study of ecological complexity following this paradigm has high potential to advance in ecology and conservation. In brief, CSS aims to discover general rules across biological, technological, social, and other types of complex systems (*6*, *21*, *40*). This broad objective has made CSS historically fluid and ever-evolving, gradually encapsulating various ideas, methods, and traditions (*5*, *41*). While CSS has some roots in ancient philosophy (e.g., Aristotle’s emergence in the *Metaphysics*), formal research into complex systems began only toward the 19th century. The scientific revolution of the 16th to 17th centuries revealed certain fundamental laws of nature, but the concept that nature can be perfectly predicted following such laws soon began to falter, particularly in micro- and cosmo-physics. For instance, in 1871, James Clerk Maxwell began to explore the limitations of the second law of thermodynamics, and, in 1890, Henri Poincaré identified strong dependence on initial conditions when predicting the motion of celestial bodies using the laws of gravitation, paving the way for chaos theory (*6*, *10*).

In the 20th century, it became increasingly clear that the global properties of complex systems can be inherently difficult to predict from the fundamental laws of nature that underlie these systems—as the adage goes, “the whole can be greater than the sum of its parts.” In turn, scientists and mathematicians began to investigate how natural laws constrain, but do not determine, the global properties of complex systems, where interactions among units can determine phenomena that emerge across hierarchical levels of complex systems (*4*, *5*, *30*, *42*–*44*). CSS embraced the need to consider interactions, providing a new paradigm for understanding reality beyond traditional scientific views. CSS developed from the conceptualization of “general systems theory,” spearheaded in the 1930s by Ludwig von Bertalanffy, by mathematical work, e.g., on self-organization and dissipative systems by Ilya Prigogine and on chaos by Edward Lorenz in the 1960s, and finally by increasing reliance on computer simulations after World War II (*45*).

Following these early developments, the study of complex systems became an explicit research focus from the 1970s (*39*), leading to the establishment of the Santa Fe Institute (https://www.santafe.edu/) in New Mexico, United States (*33*). Founded in 1984 by eight physicists, including Nobel Prize winner Murray Gell-Mann, the Santa Fe Institute was the first institution fully dedicated to research of complex systems. Since then, many centers for the study of complexity have opened across the planet. Today, the Santa Fe Institute connects a global network of scientists that are seeking a better understanding of complex systems and plays a key role in popularizing CSS within and outside of academia. Principles from CSS have been instrumental in meta-science (*46*), mathematics (*47*), physics (*29*), medicine (*48*), sociology (*49*), archeology (*50*), economy (*51*), social management (*52*), and computer science (*53*), among many other disciplines. Ecology is one of those disciplines, and it has been argued that CSS can provide important answers to many current environmental crises faced by humanity (*21*–*23*).

With the initial work in place for developing CSS, physicist Heinz Pagels suggested (in 1989) that “the nations and people who master the new sciences of complexity will become the economic, cultural, and political superpowers of the next century” (*54*). Despite skepticism that has persisted around CSS since its inception (*53*), the diffusion of this paradigm in the past three decades, together with important developments documented across many fields of knowledge (*5*, *55*), is a testament to the vision of the pioneers in this field.

## THE PHILOSOPHY OF CSS

Defining and studying “complexity” has been a long-standing challenge, not least because of different philosophical views among authors and entire disciplines. For instance, some authors categorize their object of study as either complex or not, while others conceptualize complexity along a continuum (*31*). Complexity takes on different definitions across scientific domains, e.g., computer scientists may refer to the time and computational memory required to solve a problem (*56*, *57*), whereas mathematicians may use complexity to refer to chaotic and nonlinear dynamics (*58*). It has been even suggested that complexity is “a placeholder for the unknown,” and a “nomadic term that links disparate discourses,” such that a strict definition would only be an unwarranted constraint (*32*). This flexibility might explain how some fields, such as computer science, which leverage explicit, quantitative metrics of complexity, have advanced faster than others in their quest to understand complexity. Nevertheless, the immoderate freedom of vague definitions also seems to have hindered coordination and synthesis.

CSS bypasses most of these philosophical aspects and is more operational in its definitions and implementation. Being the quantitative field that seeks to discover laws that describe phenomena in complex systems, CSS has for decades provided a robust and pragmatic framework to classify phenomena also observed in ecosystems. Ecologists turning to CSS, however, must be aware that this field demands a shift from conventional scientific paradigms (*3*, *6*, *45*, *59*). Scientists have traditionally engaged complexity following (i) determinism, i.e., the idea that systems can be explained by adequate mathematical models; (ii) reductionism, i.e., that any system composed by many entities can be understood by studying such entities individually; and (iii) disjunction, i.e., that the way to resolve cognitive problems is isolating them within specialized disciplines. These principles can fail, as for example, some systems cannot be easily predicted, even when they follow deterministic laws (*4*, *18*, *33*, *60*); the organization of units in a system can determine the emergence of some properties and the inhibition of others, hindering efforts to predict systems by studying their parts in isolation (*6*, *28*, *31*, *59*); and using interdisciplinary approaches can greatly increase our understanding of complex systems (*3*, *6*, *47*).

Because of these discrepancies, reductionist scientific approaches have often failed ecologists interested in understanding complex systems (*27*). Conversely, CSS has helped because it explicitly recognizes the importance of emergence, and it is, by nature, integrative across disciplines. Transcending reductionism is a major divide in the study of complexity between two philosophical views—restricted complexity, of which CSS is an expression, and generalized complexity (*6*, *45*).

“Restricted complexity,” in contrast to "general" or “generalized complexity,” is interested in the dynamics of complex systems composed of a large number of interacting parts (i.e., the more parts and interactions, the greater the complexity) (*61*). Restricted complexity postulates that certain phenomena make systems more difficult than others to understand and predict, bypassing epistemological and ontological considerations on complexity, and aims at understanding laws governing those phenomena. Drawing inspiration from mathematics and physics, restricted complexity emerged to address the gap left when the traditional scientific paradigm has been demonstrated to be inadequate in predicting phenomena typical of some systems (due to, e.g., chaos, nonlinearities, and tipping points). It is a search for the “laws of complexity” and may be equally prone to the paradox of following a reductionistic model as well (*33*).

Generalized complexity originated from the integration of poststructural philosophy with biology and suggests that complexity concerns not only all scientific disciplines, but also systems of knowledge. Reductionism is substituted with the seeking of a dynamic understanding of the relationship between a whole and its parts, as well as their mutual implications. Rather than the number of parts interacting in a system, generalized complexity focuses on the nature of the interactions among the parts (i.e., more complex interactions lead to more complex systems). In this view, our inference on complex systems can never be perfect because studying a system requires creating boundaries, which is at least partially an arbitrary process for complex systems, and might exclude certain aspects of the system itself and of the environment hosting that system. Note that generalized complexity does not argue against reductionism; instead, it recognizes some of its limitations.

Our analyses and approach align with the view embraced by the restricted complexity perspective, which we consider appropriate given the strong quantitative focus of modern ecology. A balance between conceptual advances and urgent action, as well as between restricted and generalized complexity perspectives, will be necessary to face the global environmental crisis (*62*, *63*).

## UNTANGLING THE FABRIC OF ECOLOGICAL COMPLEXITY

To understand how ecologists conceptualize complexity, we propose a “research weaving” exercise designed to identify general patterns in how authors conceptualize complexity in ecology (Fig. 1, B to E; see Materials and Methods) (*64*). Briefly, we first identify a set of features typical of complex systems in ecology and the environmental sciences (Table 1). We then quantify how often these features have been used in all the articles that are explicitly related to ecological complexity in the Web of Science database, and compare those to the “control” articles, which are randomly selected from ecological studies that do not refer to ecological complexity. Last, we use this dataset to describe spatiotemporal trends in the study of ecological complexity (Fig. 2), to analyze thematic diversity (Fig. 3), and to identify patterns in connections between feature usage (Fig. 4) and co-citation of the references appearing in articles that explicitly refer to ecological complexity (Fig. 5).

Because the concept of complexity should recall similar ideas for different scientists, we expect that articles explicitly referring to ecological complexity should more frequently mention features typical of complex systems than the control group articles (or “control articles”). We also predict that articles that explicitly refer to ecological complexity should be more similar among themselves than the control articles, because of ecology’s vast scope. For the same reason, we predict that patterns in how ecological complexity is conceptualized should differ across subfields of ecology, e.g., with certain features being more likely to be discussed together, and/or with some subfields citing different subsets of the literature. Support for these predictions would suggest that authors who refer to ecological complexity do so while relating to a set of shared ideas, and therefore that, at least theoretically, there is potential to organize the study of ecological complexity around the principles we identified in reviewing relevant literature in CSS (Table 1).

### Features of complex ecological systems identified from CSS

We found from the literature that scientists in CSS identified a core set of concepts that characterize complex systems. Common narratives include the idea that complexity is typical of systems composed of multiple, diverse parts and structured across different organizational levels (*3*–*5*, *18*, *21*, *33*), a vision that puts networks (*53*, *65*) and hierarchies (*9*, *66*, *67*) at the core of ecological complexity. Other concepts include spatiotemporal scale dependencies (*28*, *68*–*70*), criticality (*11*, *71*), self-organization of the parts that compose a system in increasingly sophisticated modules (*9*, *21*, *33*, *72*, *73*), and feedbacks occurring both within and between each level of the system, which stabilize and constrain both the whole system and its parts (*6*, *18*, *31*, *68*, *70*). Chaotic dynamics and the potential for alternative states, which are often contingent on the initial conditions of a system and may operate at any organizational level, complete the typical recipe of a complex system (*2*, *18*, *74*, *75*). We chose 23 representative features to synthesize more specific aspects that emerged consistently from this broad range of concepts (Table 1).

### Spatiotemporal patterns in the study of ecological complexity

We retrieved 172 articles that mention “ecological complexity” in their title or keywords. Researchers based in institutions from all continents except Antarctica contributed to this pool of manuscripts (Fig. 2A), with researchers based in North American (*n* = 266) and European (*n* = 185) institutions contributing more articles. Considering the articles mentioning “ecological complexity” in all fields (i.e., title, keywords, and abstract), we found a steady increase in research effort starting from the late 1990s, exceeding 2000 articles per year as of the end of 2021 (Fig. 2B; see also fig. S1).

### The diversity of complexity articles

We ran a topic modeling analysis using the latent dirichlet allocation (LDA) to test whether the 23 features we selected through our critical review (Table 1) are relevant to characterize complexity articles, and to what extent these contribute more to complexity than control articles. All features except “aggregation” appeared more often in the top 0.5% important features in topics from the complexity group compared to the control, and the average probability of a feature to characterize a document was higher for the complexity group (fig. S2).

Having assessed the reliability of the 23 features we identified in our critical review, we compared complexity and control articles with respect to their reference to these features. Complexity articles included a significantly (α = 0.05) higher number of features than expected from a random sample of control articles from the ecological literature (Fig. 3, A and B) and were more similar to each other than expected by chance alone (Fig. 3, C and D). Specifically, complexity articles mentioned on average 9 of 23 features, against the 6 observed in control articles (*F*_1,344_ = 86.6, *P* < 0.0001; Fig. 3A). This result was consistent when accounting for features’ relative abundances (*F*_1,344_ = 68.53, *P* < 0.0001; Fig. 3B). Regarding uniqueness, complexity articles were on average 6% more similar to each other than control articles. The average distance to the median of complexity articles was 0.51 ± 0.09, while control articles showed an average distance to the median of 0.54 ± 0.10 (*F*_1,344_ = 10.92, *P* = 0.001; Fig. 3C). For both complexity and control articles, those mentioning less than five features were typically more distant from their respective group median than the other articles, which suggests that the features mentioned in those articles were less commonly mentioned in other articles from our sample (Fig. 3D).

### A network of complexity features

The features identified in our critical review formed a highly connected network (relative connectance = 0.988; Fig. 4). Most of the features co-occurred at least once, although the features “scaling,” “interaction,” and “dynamicity” contributed disproportionately more in terms of connection strength and node weight (Fig. 4 and fig. S3). By modeling the network using an exponential random graph model (ERGM), we found that complexity articles are more likely to form connections in the network (edges) than control articles (estimate ± SE: 0.47 ± 0.02, *z* = 27.67, *P* < 0.0001). Conversely, network homophily (i.e., similar nodes are more likely to connect than dissimilar ones) was not significant (estimate ± SE: −0.04 ± 0.02, *z* = −1.91, *P* = 0.06), indicating overall that control and complexity articles tended to be interconnected with each other. Some of the most important features for the extracted network (e.g., the terms “network” and “diversity”) were not typically common to the complexity articles (Fig. 4).

### Co-citation network for the ecological complexity literature

When assessing the reference lists of all complexity articles, the Louvain clustering algorithm identified five clusters of co-citation among the top 100 most co-cited references (Fig. 5). Two clusters included 10 or fewer references and reflected the production of two research groups (Fig. 5, in gray and black). Conversely, three clusters included at least 19 references and involved several research groups. The first cluster includes, among others, the seminal work of Kuhn (*76*), Levins and Lewontin (*77*), and May (*2*), representing a tradition of basic theory, mathematics, and philosophy applied in the study of complexity (Fig. 5, in pink). The second cluster includes the work of Levin (*18*), Brown (*78*), Maurer (*79*), and Hubbell (*80*), representing a tradition of macroecological approaches and large-scale system science (Fig. 5, in blue). The third cluster includes the work of Allen and Starr (*9*), Levin (*70*), and Petrovskii *et al*. (*81*), representing a tradition of scaling approaches and application of hierarchy theory in the study of complex ecological systems (Fig. 5, in gold). Although these clusters were found when considering the 100 most cited articles, such structure remained resistant to deviations in the number of nodes in the network, except for the cluster including two seminal references by Ulanowicz (Fig. 5, in black). Overall, 68 complexity articles cited the references that determined patterns in the clusters, from which 58 cited only references from the three most important clusters. The adjacency matrix showing the pairwise co-occurrence of all 100 articles can be found in the Supplementary Materials (fig. S4).

## THEMES IN ECOLOGICAL COMPLEXITY

The concept of complexity has been historically intertwined with our understanding of nature (*3*, *32*, *33*, *82*). Many environmental challenges faced by humanity are “complex systems problems” (*13*, *14*, *16*, *19*, *21*, *22*, *24*). Solutions to these challenges might appear straightforward (e.g., reducing emissions of greenhouse gasses and halting habitat degradation), but because we lack unified theories, methods, and ultimately a comprehensive understanding of complex ecological systems, we cannot adequately assess ecosystem collapse scenarios given current and forecasted environmental conditions (*19*, *22*). Some phenomena might even be impossible to predict, a crucial aspect that scientists often fail to communicate effectively with the public (*62*). The study of ecological complexity will be central in clarifying these aspects in the coming century (*14*, *29*).

Nevertheless, our analysis suggests that the field of ecological complexity is currently disorganized, hampering a coordinated and optimized progress. The reviews that we assessed based on our preliminary literature survey (Fig. 1A and table S1) focus on a broad spectrum of unrelated themes, and we could not assess complexity articles concerning CSS independently, because we only found 24 such articles. Furthermore, ecology and conservation are lagging behind recent developments in complexity science (*22*, *58*, *72*) despite increasing numbers of articles on the topic. For instance, a recent analysis suggests that deterministic chaos might not be uncommon in nature (*83*) but attempts to reveal its influence on natural systems remain comparatively rare. Similarly, the potential for catastrophic scenarios is largely understudied (*19*); meanwhile, recent evidence suggests that global warming is likely to trigger climatic tipping points (*15*). These are dynamics that must be understood urgently, a goal that could be directly pursued following principles from CSS. In the following sections, we discuss how we could best achieve this objective.

### What makes a system complex?

Given that complexity has been studied as an attribute of ecological systems (*39*, *75*, *84*, *85*), it is theoretically possible to identify features that make some systems more complex than others. We therefore conducted a critical review to identify features typical of complex systems as described in the CSS literature. Through this exercise, we reduced very broad, interconnected aspects of complexity into a more tractable set of features typical of complex systems (Table 1). Our synthesis goes beyond applications within specific subfields and encompasses a broad range of perspectives, following both seminal references (*3*–*6*, *18*, *21*, *33*, *43*, *71*, *86*–*89*) and more recent work that focuses on application of the CSS paradigm in ecology and conservation (*10*, *11*, *31*, *38*, *55*, *58*, *68*, *72*, *75*, *84*, *90*–*92*). Therefore, we suggest that Table 1 can be used as a template to organize the study of complex ecological systems around well-established themes in CSS.

We recognize that there are elements of subjectivity in our work. Furthermore, our analysis neglects some concepts in ecological complexity due to the approach that we used, and future perspectives should therefore expand on concepts that we have not included here. For instance, we omitted some concepts developed in CSS from our list of features, including panarchy (*93*), heterarchy (*92*), brittleness (*94*), and criticality (*11*, *71*). These are important conceptual aspects of CSS but are less general than the features we selected, e.g., they rarely occur in the complexity papers we retrieved (Fig. 1, C and D, and fig. S5). Nevertheless, perhaps oversimplistically, the concepts embodied by these terms can be represented by combining different features proposed in our synthesis. For instance, “panarchy” relates to stability and dynamicity, “heterarchy” to networks and hierarchies, “brittleness” to resilience and modularity, and “criticality” to dynamicity, fractality, scaling, and attractors (Table 1). We also purposely chose to represent some of our features using very general terms—for example, the feature “diversity,” with the term “biodiversity” alone being the object of volumes of discussion (*95*). Another relevant example is scaling, which has been used loosely to describe the property of some ecological phenomena to change across spatial scales (*96*, *97*), and more formally in the context of scale-invariant laws discovered in ecology (*11*) (see discussion of “efficient theories” below). Keeping these limitations in mind, we believe that the flexibility coming with the broad terms we chose will accommodate the many different phenomena described in complex ecological systems under a broad, but organized, conceptual framework. Our review is not a definitive guide to the vast field of ecological complexity but is a starting point of an effort to explore CSS for ecologists interested in complexity.

While we recognize that some of our methodological choices may be somewhat arbitrary, it is not clear that there is an objective way to reproduce this study while entirely removing personal evaluations. Relying solely on bibliometric tools to identify alternative features to those we propose would have substantial limitations (*98*). For instance, the same terminology can be used by different authors to express different concepts [see, e.g., “complex adaptive systems” sensu (*3*) versus (*4*)], and while the human mind can recognize these patterns, algorithms would likely fail to do so. Furthermore, text analysis would skew our assessments toward concepts in peer-reviewed papers, neglecting books, letters, lectures, and personal communications that we have used to inform our assessment (*99*). For this reason, we preferred a critical review to a topic modeling approach for identifying the features synthesized in Table 1. Acknowledging these aspects of our work, we next discuss how we used the template of 23 features to assess how ecological complexity has been conceptualized in the peer-reviewed literature.

### How is ecological complexity discussed in the literature?

Our analyses found that the number of articles referring to “ecological complexity” has increased exponentially in the past 50 years (Fig. 2 and fig. S1), mirroring the trend observed for articles that refer more broadly to “complexity” (and involving all continents except for Antarctica). Despite this growth, what authors conceptualize when referring to ecological complexity has remained largely unanalyzed. In parallel to reviewing CSS in relation to ecological systems (Fig. 1, B and D), we provide a quantitative assessment of how authors have conceptualized ecological complexity in relation to the features identified in our critical review (Fig. 1, C to E, and Table 1).

Overall, we found limited differences between complexity and control articles. For instance, approximately a quarter of the complexity articles mentioned fewer features than the average control article, and complexity articles were only 6% more similar to each other than control articles (Fig. 3). The term complexity seems therefore to have been often used loosely, confirming the perspective that the word “complexity” is often used as a synonym for “complicated,” or as a “placeholder for the unknown” (*32*). This result suggests that many articles refer to ecological complexity inconsistently, but while invoking pivotal concepts in complexity science, or that these articles focus on a few of the features typical of complex systems, rather than covering multiple aspects discovered in our review. Similarly, assessing the co-occurrence of features revealed a highly connected network, with little defining structure and 98% of all possible connections fulfilled (Fig. 4). Last, only about a third of the complexity articles contributed to the 100 most co-cited references (Fig. 5). Together, these parallel lines of evidence suggest that the study of ecological complexity has lacked coordination and structure.

One could argue that the true essence of ecological complexity is not captured by the features revealed in our review (Table 1). However, we identified meaningful patterns that suggest the contrary. For instance, a significantly higher number of features in complexity articles indicates that authors appealing to ecological complexity might agree that more complex systems are complex owing to the interplay of a larger set of features. Furthermore, ~60% of the features identified in our review were significantly more likely to be related to complexity articles (14 of 23 features; Fig. 4), with this number increasing to ~80% of the features (19 of 23 features) when assessing the presence of features rather than frequency of use. A caveat to these results is that not all analyses engaging with complexity require consideration of many features (e.g., macroecological models), and unnecessarily complicated models are inconsistent with principles from CSS. Finally, our analysis identified expected relationships based on current ecological theory, such as those between scales and hierarchies (*67*), and networks and interactions (*65*).

Most notably, the results of co-citation network analysis are consistent with three prominent philosophies in ecology (Fig. 5). The first co-citation cluster emerged from literature referencing complexity in relation to a long tradition of basic theory and mathematics (*1*, *2*, *31*, *76*). The second co-citation cluster emerged from literature that refers to complexity in relation to the concepts of scales and hierarchies (*9*, *38*, *67*, *70*, *100*). The third co-citation cluster emerged from literature that invokes complexity in relation to macroecological theory and the study of large-scale systems, or those containing many data points describing individual objects in a system (*79*, *80*, *101*–*103*). These schools of thought have been prominent in ecology for decades and will likely continue to be so. Recent developments suggest that the role of theory in ecology will be crucial in the era of big data (*104*), that scales can be a mediator of seemingly irreconcilable ecological patterns (*105*), and that a macroecological approach might be our only way to escape local contingencies in the pursuit of generality (*28*).

Ultimately, imprecision in the use of the term complexity in the ecological literature means that descriptions of it are not converging on a single set of shared understanding. Despite this and some limitations of text mining approaches, we found promising trends for coordination of research efforts at the interface between ecology, conservation, and CSS.

## TOWARD A COHESIVE UNDERSTANDING OF ECOLOGICAL COMPLEXITY

We interpret the results of our research weaving exercise as evidence that studies targeting complexity in ecology would benefit from following principles developed in CSS. This could be a key direction for ecological research because such studies have the potential not only to reveal how ecological systems are responding to global change, but also to advance theory in both disciplines. On the one hand, developments in CSS can provide ecology with innovative theories and tools. For instance, studies on the mathematics of fractals and of self-similarity permeate many fundamental theories in ecology (*106*, *107*); mechanistic simulations such as cellular automata and agent-based models, developed by computer scientists in the 1950s, are increasingly used to explore emergent biological phenomena (*71*, *108*, *109*); and genetic algorithms (*86*) are now routinely used for ecological applications (*110*). Relatedly, ecology has held a special place in the development of theory for CSS. Research on populations and ecosystems has provided many insights, e.g., on nonlinear dynamics (*111*, *112*), chaos (*60*, *74*, *83*), tipping points (*12*, *15*, *113*), scaling (*11*, *70*, *114*), resilience (*115*, *116*), and natural computation (*58*). Better integration of principles from CSS in ecology will reinforce this virtuous cycle.

An example of successful integration between CSS and ecology comes from the application of principles from the three clusters outlined by our analysis—theory, scaling, and macroecology (Fig. 5)—in the search for efficient theories [sensu (*104*)]. These are typically “theories of averages” that identify regularities appearing in ecological systems at certain levels of organization (*11*, *21*). Efficient theories generate first-principle predictions across scales of ecological organization, usually based on mathematical models, and ecologists have successfully dealt with the complexity of ecological systems by developing a number of these theories. Ecological theories developed in the past decades allow testing explicit predictions on a variety of phenomena including biodiversity (*106*, *117*, *118*), abundances and spatial distributions of species (*118*), distribution networks in animals and plants (*107*), or responses to temperature across levels of biological organization (*119*). The principles on which these theories are based include information theory (*120*, *121*), optimization of energy dissipation (*107*), metabolic rates (*122*), or chemical laws (*119*). While it has been argued that identifying such efficient theories should be the primary goal of ecologists (*62*), progress is slow, in part because of the urgency to solve pressing environmental issues with other tools currently available (*63*).

This tension between a search for general, theoretical advancements, and resolving more specific case studies, is useful to advancing ecology. There is no doubt that traditional scientific views will continue to provide important insights on ecological systems, yet approaches from CSS have already yielded fresh perspectives on historical dilemmas that could not be solved with traditional approaches. These include insights on the stability-diversity relationship [e.g., negative feedbacks in species interactions can promote stability in dynamic systems (*123*)], on critical thresholds in habitat loss and fragmentation [e.g., genetic drift can depend on thresholds of habitat area left in a landscape (*124*)], on the evolution of maladaptive characters [e.g., when considering spatial dynamics, maladaptive traits can be retained in a population despite their disadvantages (*125*)], and on the regulation of emergent behaviors [e.g., simple rules can explain how fireflies coordinate their light pulses (*126*)], among many more topics (*58*, *72*).

A central message of our work is that developments in CSS will lead to developments in ecology and conservation (and vice versa) only if ecologists will conceptualize ecological “complexity” with more clarity and depth. We propose two simple principles that will help to this end. First, it is always desirable to specify what exactly one means when referring to complexity. While working on our critical review, we noticed that definitions of ecological complexity are extremely rare in the literature, with “complexity” being sometimes used as a buzzword (*127*). We therefore propose that the term complexity in ecology should be used carefully by studies that are not assessing ecological systems through the lenses of CSS. Second, attempts to measure the complexity of natural systems are very common [e.g., (*39*, *84*, *85*, *128*)], and we believe that these efforts could often be sharpened. When measuring properties of systems and referring to those as metrics of complexity, authors could first refer explicitly to the phenomenon that a metric represents, and then discuss their results in relation to ecological complexity, rather than conflating the two aspects. We provide a nonexhaustive list of metrics used to measure complexity as an example (Table 2), specifying the relationships among these metrics and the features identified by our review. Many questions in ecology and conservation can be answered without appealing to concepts and approaches from CSS; for those studies, we suggest that referring to complexity only increases confusion in an already difficult field.

**Table 2.** Popular metrics characterizing complexity features. A non-exhaustive list of metrics used in the ecological literature when assessing ecological complexity and their relationship with the features identified in our article. We refer particularly to (*39*, *65*, *75*, *84*, *85*, *128*) for comprehensive reviews of metrics designed to measure complexity.

Feature	Metric	Reference
Chaos	Lyapunov exponent. It represents the rate of separation of infinitesimally close trajectories, measuring how a dynamic system is sensitive to initial conditions.	(*153*)
Diversity	Shannon entropy: −∑*_i_P*(*x_i_*) log *P*(*x_i_*), where *P* is the probability of an event *i*. Measures the amount of information in an event drawn from that distribution.	(*75*)
Diversity	Mean information gain: *H*_s_(*L* + 1) − *H*_s_(*L*), where *H*_s_ is the Shannon entropy of the sequence of length *L.* Measures the amount of information gained by knowing an additional step in time or space.	(*84*)
Diversity	Fluctuation complexity: $\sum_{i,j}P_{L,ij}\log{\left(\frac{P_{L,i}}{P_{L,j}}\right)}^{2}$, where *P*_*L,ij*_ is the probability of observing *j* immediately following *i*. Measures the degree of structure in a time series.	(*84*)
Dynamicity	Information theoretic measure of correlation between the two halves of a stochastic process lim_t → ∞_ *I*(*X*_−*t*_*X*_−*t*+1_…*X*_−1_; *X*_0_*X*_1_…*X_t_*). Also known as effective measure complexity, predictive information, and excess entropy.	(*154*)
Fractality	Fractal dimension: log(*N*) /log(*r*), where *N* is the number of self-similar pieces and *r* is a magnification factor. Measures the degree of self-similarity.	(*84*)
Fractality	Power law: *P*(*x*) = *cx*^−γ^. Measures the degree of pattern consistency across scales.	(*155*)
Network	Modularity: *Q* = ∑*_i_*[*e_ij_* − (∑*_j_e_ij_*)^2^], where *e_ij_* are the fraction of edges that link nodes in cluster *i* to nodes in cluster *j*. Measures the strength of division of a network into groups (modules).	(*65*)
Network	Connectance: the proportion of realized ecological interactions (*m*) among the potential ones (*L*), or *L/m*. Potential links are most often calculated as the squared species richness. Measures the fraction of all possible links that are realized in a network.	(*65*)
Network	Degree distribution: the distribution (*P_k_*) of the number of links (interactions) per species; if *N*(*k*) is the number of nodes with *k* interactions, and *S* is the total number of species in the network, then *P*(*k*) = *N*(*k*)/*S*. Measures the heterogeneity of a system: If all the nodes have the same degree *k*, the network is completely homogeneous.	(*65*)
Network	Singular value decomposition (SVD) entropy: within a matrix *i*, the nonzero singular values (σ*_i_*) and the number of nonzero entries (*k*) are extracted. SVD entropy is then calculated as: $J=\frac{-1}{ln(k)}\sum_{i=1}^{k}s_{i}\times ln(s_{i})$ where *s_i_* = σ*_i_*/sum(σ). Measures the number of vectors needed for an adequate explanation of the dataset, where higher values indicate that the dataset cannot be efficiently compressed.	(*156*)
Stability	Eigenvalues of the Jacobian matrix: [*J_ij_*] = [∂*f*_*i*_/*∂x_j_*], where *x* is a state and *f_i_* = *dx_i_*/*dt* at a fixed point. If all real parts of the eigenvalues are negative, this fixed point is a stable attractor, and the system returns to the steady state after perturbation.	(*128*)
Stability	Coefficient of variation: CV = σ/μ, where σ is the standard deviation and μ is the average of a time series. Measures the level of dispersion around the mean of a series.	(*152*)
Self-organization	Mutual information: measures the difference in uncertainty between the sum of the individual random variable (example, *X* and *Y*) distributions and the joint distribution: *I*(*X*;*Y*) = *H*(*X*) + *H*(*Y*) − *H*(*X*,*Y*), where *H* represents Shannon entropy. When two variables are completely independent from one another, *H*(*X*) + *H*(*Y*) = *H*(*X*,*Y*) and the mutual information is zero. Any covariance between *X* and *Y* (i.e., self-organization or order) will result in an uncertainty in the joint distribution that is lower than the sum of their individual distributions.	(*128*)

## THE ROLE OF ECOLOGICAL COMPLEXITY IN AN AGE OF URGENCY

One of the most important lessons learned from the development of CSS is that, with a proper focus, we can estimate and even predict how global environmental change will affect many types of complex systems (*20*). Embracing ideas and approaches from CSS is, therefore, more urgent than ever as we attempt to understand how anthropogenic, global change will affect our planet. As we write, Earth has experienced another season of records in climatic anomalies (*129*). The summer of 2022 was the hottest recorded in the history of Europe, as much as China has experienced the longest heatwave ever recorded. Several rivers and bodies of waters worldwide are experiencing reductions in discharge and area due to drought, including the Po, Rhine, and Loire rivers in Europe, the Colorado river in North America, and China’s largest freshwater body, Poyang Lake. Africa was affected by the worst drought in 70 years, while intensifying wildfires are causing extreme damage to ecosystems and human societies on many different continents (*130*). These phenomena are affecting not only biodiversity and ecosystem functions but also the supply of energy and primary services (e.g., water) to millions of people (*23*). CSS provides a relevant conceptual framework to assess ongoing environmental crises not only because of the conceptual advancements outlined in this review (*21*) but also because it recognizes the tight links connecting ecosystems and human societies into social-political-ecological entities (*103*), embracing the important roles of sustainability, governance, politics, and ethics for applied biodiversity conservation in the face of global environmental change (*23*, *27*). This holistic understanding of environmental and social issues will be necessary as we embark in a critical transition to more sustainable and ethical societies, in an attempt to mitigate the effects of human activities on Earth (*23*, *25*, *26*).

Ultimately, while we primarily provide guidelines to integrate CSS, ecology, and conservation, our hope is that this work will also promote the pursuit of consilience and integration with social aspects of the modern environmental sciences. Reflecting on how we study and refer to ecological complexity has great potential to stimulate the sharing of ideas from areas that likely have already participated in CSS, but whose contributions remain poorly known to the Western science. These efforts will benefit from inclusion of perspectives from underrepresented regions (*3*, *6*), such as the Global South, which remains marginalized in the study of ecological complexity (Fig. 2A). Likewise, maximizing collaborations beyond the limited scope of one’s own research group and promoting international collaborations across country borders will be a key step to bring unexplored ideas and hypotheses into CSS (*131*). In an age of globalization and potentially catastrophic environmental changes, embracing the principles of CSS—including being open to original, transdisciplinary ideas—has never been more timely.

## MATERIALS AND METHODS

### Overview

We prepared and analyzed a dataset to assess how often the features typical of complex systems are used in the literature referring to complexity in ecology (Fig. 1). This required first to identify features typical of ecological complexity (Table 1). Next, through a systematic literature survey, we sourced two bodies of literature: a treatment (i.e., articles using the term “ecological complexity” in their title; hereafter complexity articles) and a control group (i.e., general articles in ecology; hereafter control articles) (Fig. 1C). Afterward, we quantified the use of the selected features in control and complexity articles (Fig. 1, B to E). The analysis followed four steps: (i) describing general patterns in complexity articles, (ii) comparing the diversity of features in complexity versus control articles, (iii) exploring the relationships among complexity features within complexity articles, and (iv) identifying influential references in ecological complexity literature. We ran all analyses in R v.4.1.2 (*132*), using the “tidyverse” suite v.1.3.1 (*133*) for data wrangling and visualizations. We refer readers to the Data and materials availability statement for information on scripts and data used in this study. Our analysis is based on the premise that complexity is an attribute of ecological systems, and thus that we can identify properties of systems that are typically associated with the idea of complexity (*84*). This perspective relates to the paradigm of restricted complexity and allows us to quantitatively assess the ecological literature (see the “The philosophy of CSS” section).

### Data preparation

#### Identifying features typical of ecological complexity

We began by compiling a list of features that are typically associated with the study of complexity in the scientific literature, with an emphasis on ecological literature. An initial screening showed that different articles that mention and define complexity highlight different features (table S1). For instance, we tried searching for reviews summarizing ideas from complexity science in ecology with little success [but see (*68*, *72*)]. We concluded that identifying the features typical of complex systems in ecology as described in complexity science was not possible based on an automatic procedure. This is because different authors use complexity to describe very different ideas and processes or use different words to refer to the same concept, which makes the design of a systematic review prohibitive. We therefore chose an unstructured, critical review approach (*134*), based on a mixture of article retrieval with fixed search strings (e.g., “complexity” AND “ecology” AND “review”) and scouting of the references cited in seminal articles that we deemed relevant for our exercise.

We refer to several documents for discussion of the features identified in our review (Table 1). These include books and book chapters (*3*, *5*, *6*, *21*, *43*, *58*, *86*–*88*), and various types of peer-reviewed scientific articles (hereafter “articles”), particularly reviews (*4*, *10*, *11*, *18*, *31*, *33*, *38*, *55*, *68*, *71*, *72*, *75*, *84*, *89*–*92*). While other relevant perspectives certainly exist in the literature, we suggest that this body of literature captured what characterizes complex systems reasonably well because we targeted the perspective of several independent groups of authors interested in CSS, often recognized as leaders in the study of complexity, and because we included recent reviews, capturing ideas at the forefront of the study of ecological complexity.

Our critical review identified 23 major features typical of ecological complexity (Table 1). We note that some features initially under consideration, including the terms “hysteresis,” “panarchy,” and “heterarchy,” were removed because they appeared rarely in the articles assessed in our analysis (fig. S5). We used single words to represent each of the selected features, aiming to ensure comparability on the frequency of use of different features across studies (Table 1). These words were carefully chosen to be as broadly representative of the features as possible. For example, a common feature emerging in the literature is the idea that complex systems are composed of units that differ among themselves; this is typically discussed as “diversity,” but can also be associated with “entropy,” e.g., in biodiversity science, and “heterogeneity,” e.g., in landscape ecology. Because we selected a single word to represent each of the compiled features to ensure comparability in features’ counts among articles, we acknowledge that our results might be sensitive to the word selected. We also recognize that any two articles might share similar features but address them with different approaches. These nuances are challenging to capture when conducting bibliometric analyses, and our results should be evaluated keeping this in mind.

#### Systematic mapping of the literature

Next, we retrieved articles representing research on ecological complexity to compare them with more general articles in the field of ecology. This was carried out through literature searches on the Web of Science Core Collection database over all the citation indices, all document types, and all years (exploratory queries between May and July 2021; final query on 23 September 2021). In an exploratory scoping phase, we trialed different search terms by running searches and considering the relevance of the first references. We found that using overly broad terms (e.g., <ALL = “ecology” AND “complexity”>) yielded a large number of articles (*n* > 14,000). On the opposite end, incorporating specific terms typically associated with ecological complexity either matched a limited number of articles (e.g., “homeostasis”) or captured several articles not relevant to the question posed (e.g., the term “network” generated many articles on energy infrastructure). We found a balance between specificity and quantity by searching for general terms but restricting the search to the title (TI) and keywords (AK). The final query was <TI = “ecolog* complex*” OR AK = “ecolog* complex*”>, which returned 188 results (henceforward “complexity” articles; Fig. 1C). We assumed these articles to be a random sample of literature that generally refer to complexity in ecology and the environmental sciences, i.e., that the study of “ecological complexity” is not an independent avenue of research from the broader study of complexity in ecology. As a control (henceforward “control” articles), we randomly selected 188 articles from the ecological literature, using the query <WC = “Ecology” NOT (TI = “ecolog* complex*” OR AK = “ecolog* complex*”>, where WC is used for searching through the Web of Science categories (Fig. 1C). In all analyses, we looked at differences between complexity and control articles to understand if complexity articles were more consistent with CSS literature (Fig. 1, D and E).

#### Text mining

The last step of our dataset preparation was to quantify how often each of the features listed in Table 1 occurred in each article. We did this by performing text mining analyses on the full-text file of each of the articles returned by our searches. We first downloaded all full-text files as .pdf files and extracted their text using the package “pdftools” v.3.1.0 (*135*). Because we could not retrieve 24 files (16 complexity and 8 control articles), the final sample size for the text mining analysis was 172 complexity articles and 180 control articles. Once we extracted the text from the articles, we screened them to obtain all the n-grams (strings of one or more adjacent words; henceforth “words”) within each article using the package “tidytext” v.0.3.2 (*136*) and “stringr” v.1.4.0 (*137*). Some of the features could be found either as single or composite words (Table 1); thus, we extracted both unigrams and bigrams from articles using strings compatible with both British and American spellings. For single words (e.g., “scale”), we cross-referenced the string with the unigrams extracted from the text (i.e., every single word in the article). For two-part words (e.g., “self-organization”), we cross-referenced the search string with all bigrams extracted from the text (i.e., every combination of two consecutive words). For the features that could be found either as single, hyphenated, or two-part words (e.g., “nonlinear” versus “non-linear” versus “non linear”), we cross-referenced the strings separately using both approaches. Last, we summed the results from the cross-reference to determine the total number of times each feature appeared in each article and to calculate the relative frequency of each feature as the ratio between the number of uses of a given feature and the total number of words in that article. We note that four control and two one-page-long complexity articles did not include any features from Table 1.

### Analysis

#### Spatiotemporal patterns in the study of complexity

The first set of analyses was aimed at describing general patterns in complexity articles. We assessed the number of complexity articles published each year up to 2020 to determine whether research effort increased over time. We also extracted the affiliation of all authors from each article to investigate whether the collaborations were carried out nationally or internationally, and how these were globally distributed. We automatically retrieved the geographic coordinates for each affiliation using the package “ggmap” v.3.0.0 (*138*).

#### Topic modeling

We ran a topic modeling analysis using the LDA method (*139*) to verify whether the 23 features we selected through the critical review (Table 1) are meaningful to describe ecological complexity. LDA assumes that text documents are a mixture of topics, and topics are composed of a mixture of words (with individual words having differential probabilities of associating to a given topic). LDA is a mathematical method for finding the mixture of words that is associated with each topic while also determining the mixture of topics that describes each document. First, we extracted the full text of all articles in the complexity and control groups and preprocessed the text (e.g., removed stop words and punctuation, combined hyphenated words, and singularized all words). Next, we ran an LDA on the preprocessed text of all articles with the function LDA in the R package “topicmodels” v.0.2.12 (*140*), setting the number of topics to 100 and using the variational expectation-maximization algorithm. We then extracted the per-topic-per-word probability for each word (beta parameter; fig. S2). Because LDA provides near-zero probability for most of the words in topics, we selected only the 0.5% highest probabilities of our data in each topic by taking only the values above the upper limit of the 0.99 highest density interval (HDI) of our posterior distribution. Afterward, we ranked the beta values of each word within each topic and grouped them based on whether they ranked at the lowest (Q1) or the highest quantile (Q4). If the probability and frequency of a feature was higher in the complexity group, we considered it to be more important in characterizing this group.

#### The diversity of complexity articles

To compare complexity and control articles, we ran a series of analyses inspired by classical community-level biodiversity analyses. In these analyses, we treated each complexity feature as a “species,” and each article as a “site.” We calculated feature richness (i.e., number of features discussed in each article) and the effective number of features of first order [i.e., exponential of the Shannon entropy calculated using the relative frequency of features used in each paper; (*141*)] to evaluate whether complexity articles tend to encompass more of the features typical of ecological complexity compared to control articles. Given how we delimited the terms associated with complexity, we assumed that articles referring to more features should generally capture the idea of complexity better (but see the “How is ecological complexity discussed in the literature?” section for discussion of caveats).

Additionally, we assessed the uniqueness of the features in each complexity and control article by analyzing the multivariate homogeneity of group dispersion (PERMDISP), as calculated using the package “vegan” v.2.5.7 (*142*). A common measure of multivariate dispersion (i.e., variance) for a group of samples (i.e., articles) is to calculate the average distance of group members (i.e., control versus complexity articles) to their spatial median, and test if the dispersions are different with analysis of variance. PERMDISP requires a symmetrical matrix of dissimilarities between pairs of articles, which we calculated using the Bray-Curtis dissimilarity metric applied to each feature relative frequency. Last, we tested which features were typical of complexity or control articles using an indicator species analysis with “indicspecies” v.1.7.9 (*143*).

#### Network of complexity features

We explored relationships among the complexity features using a network approach. Specifically, we constructed a bipartite (i.e., containing two node types) directed network to link complexity articles with the features retrieved from our review (Table 1). In this network, the first node type represents individual articles, and the second node type represents the features. We weighted the edges connecting the two node types in the bipartite network by the relative usage of each feature within each article. Once we constructed the bipartite network, we projected it as a single mode or “unipartite” network for ease of visualization and analysis. In the unipartite network, all nodes are treated as the same type and directionality is lost. We calculated the importance of each node in the network as the sum of the edge weights of the adjacent edges of the node (henceforth “strength”). We also estimated realized connectance (RC), namely, the proportion of possible links between nodes that are realized, as

$$\mathrm{RC}=L\left[\frac{2}{S(S-1)}\right]$$

where *S* represents the number of nodes and *L* is the actual number of links realized among all the nodes in the network. To estimate the degree of discrepancy between article types, we tested the probability of connection between complexity and control articles within the network by using ERGM (*144*). In ERGMs, *Y_ij_* designates the probability of forming a link between articles *i* and *j*, with *Y_ij_* = 1 if there is a network link, and *Y_ij_* = 0 otherwise. Each value *y_ij_* specifies the observed value *Y_ij_* in a system governed by a matrix of predictor variables **Y** and links **y**—i.e., the network. The general form of ERGM can be derived as follows

$$\mathrm{Pr}(Y=y)=\frac{\exp[\theta^{\prime }g(y)]}{k(\theta )}$$

ERGMs assume that the structure of a graph can be explained by a vector of network statistics *g*(*y*) relating to network configuration, and to model parameters θ associated with *g*(*y*). The normalization term *k*(θ) ensures that probabilities sum to 1. Note that *g*(*y*) can be interpreted as covariates in a model that predicts edge occurrence, and that, here, it represents network homophily, i.e., the degree to which nodes are connected based on similarity of their attributes. For this analysis, we constructed a bipartite incidence network, starting from an incidence matrix that included both complexity and control articles. We projected the network to visualize the connections among articles through the features used. The projected network was introduced as a response variable in an ERGM fitted using the package “ergm” v.4.1.2 (*145*–*147*), with the formula (in *R* notation)

$$\begin{array}{rl} \mathrm{Network}∼ & \;\mathrm{link}\;+\mathrm{nodeMatch}\;(\text{″}\mathrm{Group}\text{″})\\ & +\mathrm{nodeFactor}\;(\text{″}\mathrm{Group}\text{″}) \end{array}$$

where “Group” is a categorical variable discriminating complexity and control articles, nodeMatch tests network homophily in terms of article type, and nodeFactor tests the overall probability of nodes forming a link based on their article type.

#### Network of co-citations

We extracted the reference list from all complexity articles and used it to build a co-citation network, seeking to identify broad trends within the study of ecological complexity. Co-citation networks describe the number of times a reference was cited alongside others, and how often these were co-occurring in the reference lists. Analysis of co-citation networks has been proposed as a tool to enhance transdisciplinary research because it allows identifying key articles that act as bridges between (sub)disciplines, as well as groups of authors focusing on similar research topics (*148*, *149*). We identify these clusters in an unsupervised way using a Louvain clustering optimization, a greedy optimization algorithm often used in network analyses due to its fast computation time and performance (*150*). This way, we let clusters emerge without imposing a fixed number of clusters a priori.
