# Supplementary material for: Toward a cohesive understanding of ecological complexity
Source: Sci Adv. 2023 Jun 21;9(25):eabq4207. doi: 10.1126/sciadv.abq4207 (PMC10284553; doi:10.1126/sciadv.abq4207)
Supplement: Supplementary file 1 — Table S1 Figs. S1 to S5 [file sciadv.abq4207_sm.pdf]

Science Advances
AAAS

Supplementary Materials for

Toward a cohesive understanding of ecological complexity

Federico Riva *et al.*

Corresponding author: Federico Riva, federico.riva.1@unil.ch; Caio Graco-Roza, caio.roza@helsinki.fi

Sci. Adv. **9**, eabq4207 (2023)
DOI: 10.1126/sciadv.abq4207

This PDF file includes:

Table S1
Figs. S1 to S5

Table S1.

List of review studies retrieved by the search on the Web of Science using the word “Complexity” in the “Ecology” and “Environmental Sciences” categories. The original search retrieved 23,703 manuscripts published between 2000 and 2021 (search conducted on July 14th, 2021), from which 71 were review studies.

Authors	Article Title	Source Title	Publication Year	DOI
Kappeler, PM	A framework for studying social complexity	BEHAVIORAL ECOLOGY AND SOCIOBIOLOGY	2019	10.1007/s00265-018-2601-8
Merow, C; Smith, MJ; Edwards, TC; Guisan, A; McMahon, SM; Normand, S; Thuiller, W; Wuest, RO; Zimmermann, NE; Elith, J	What do we gain from simplicity versus complexity in species distribution models?	ECOGRAPHY	2014	10.1111/ecog.00845
Chaplin-Kramer, R; O'Rourke, ME; Blitzer, EJ; Kremen, C	A meta-analysis of crop pest and natural enemy response to landscape complexity	ECOLOGY LETTERS	2011	10.1111/j.1461-0248.2011.01642.x
Donohue, I; Hillebrand, H; Montoya, JM; Petchey, OL; Pimm, SL; Fowler, MS; Healy, K; Jackson, AL; Lurgi, M; McClean, D; O'Connor, NE; O'Gorman, EJ; Yang, Q	Navigating the complexity of ecological stability	ECOLOGY LETTERS	2016	10.1111/ele.12648
He, P; Maldonado-Chaparro,	The role of habitat configuration	BEHAVIORAL ECOLOGY AND	2019	10.1007/s00265-018-2602-7

AA; Farine, DR	in shaping social structure: a gap in studies of animal social complexity	SOCIOBIOLOGY		
Tuck, SL; Winqvist, C; Mota, F; Ahnstrom, J; Turnbull, LA; Bengtsson, J	Land-use intensity and the effects of organic farming on biodiversity: a hierarchical meta-analysis	JOURNAL OF APPLIED ECOLOGY	2014	10.1111/1365-2664.12219
Arnosti, C; Wietz, M; Brinkhoff, T; Hehemann, JH; Probandt, D; Zeugner, L; Amann, R	The Biogeochemistry of Marine Polysaccharides: Sources, Inventories, and Bacterial Drivers of the Carbohydrate Cycle	ANNUAL REVIEW OF MARINE SCIENCE, VOL 13, 2021	2021	10.1146/annurev-marine-032020-012810
Parrish, B; Heptonstall, P; Gross, R; Sovacool, BK	A systematic review of motivations, enablers and barriers for consumer engagement with residential demand response	ENERGY POLICY	2020	10.1016/j.enpol.2019.111221
Vila, M; Espinar, JL; Hejda, M; Hulme, PE; Jarosik, V; Maron, JL; Pergl, J; Schaffner, U; Sun, Y; Pysek, P	Ecological impacts of invasive alien plants: a meta-analysis of their effects on species, communities and ecosystems	ECOLOGY LETTERS	2011	10.1111/j.1461-0248.2011.01628.x

Sheriff, MJ; Peacor, SD; Hawlena, D; Thaker, M	Non-consumptive predator effects on prey population size: A dearth of evidence	JOURNAL OF ANIMAL ECOLOGY	2020	10.1111/1365-2656.13213
Casewell, NR; Wuster, W; Vonk, FJ; Harrison, RA; Fry, BG	Complex cocktails: the evolutionary novelty of venoms	TRENDS IN ECOLOGY & EVOLUTION	2013	10.1016/j.tree.2012.10.020
Brack, W; Ait-Aissa, S; Burgess, RM; Busch, W; Creusot, N; Di Paolo, C; Escher, BI; Hewitt, LM; Hilscherova, K; Hollender, J; Hollert, H; Jonker, W; Kool, J; Lamoree, M; Muschket, M; Neumann, S; Rostkowski, P; Ruttkies, C; Schollee, J; Schymanski, EL; Schulze, T; Seiler, TB; Tindall, AJ; Umbuzeiro, GD; Vrana, B; Krauss, M	Effect-directed analysis supporting monitoring of aquatic environments - An in-depth overview	SCIENCE OF THE TOTAL ENVIRONMENT	2016	10.1016/j.scitotenv.2015.11.102
Sterner, T; Barbier, EB; Bateman, I; van den Bijgaart, I; Crepin, AS; Edenhofer, O; Fischer,	Policy design for the Anthropocene	NATURE SUSTAINABILITY	2019	10.1038/s41893-018-0194-x

C; Habla, W; Hassler, J; Johansson-Stenman, O; Lange, A; Polasky, S; Rockstrom, J; Smith, HG; Steffen, W; Wagner, G; Wilen, JE; Alpiza, F; Azar, C; Carless, D; Chavez, C; Corial, J; Engstrom, G; Jagers, SC; Kohlin, G; Lofgren, A; Pleijel, H; Robinson, A				
Carmona, CP; de Bello, F; Mason, NWH; Leps, J	Traits Without Borders: Integrating Functional Diversity Across Scales	TRENDS IN ECOLOGY & EVOLUTION	2016	10.1016/j.tree.2016.02.003
Sundqvist, MK; Sanders, NJ; Wardle, DA	Community and Ecosystem Responses to Elevational Gradients: Processes, Mechanisms, and Insights for Global Change	ANNUAL REVIEW OF ECOLOGY, EVOLUTION, AND SYSTEMATICS, VOL 44	2013	10.1146/annurev-ecolsys-110512-135750
Symonds, MRE; Moussalli, A	A brief guide to model selection, multimodel inference and model averaging in behavioural ecology	BEHAVIORAL ECOLOGY AND SOCIOBIOLOGY	2011	10.1007/s00265-010-1037-6

	using Akaike's information criterion			
Fino, D; Bensaid, S; Piumetti, M; Russo, N	A review on the catalytic combustion of soot in Diesel particulate filters for automotive applications: From powder catalysts to structured reactors	APPLIED CATALYSIS A-GENERAL	2016	10.1016/j.apcata.2015.10.016
Kim, KH; Kabir, E; Jahan, SA	Airborne bioaerosols and their impact on human health	JOURNAL OF ENVIRONMENTAL SCIENCES	2018	10.1016/j.jes.2017.08.027
Qiu, RJ; Lin, M; Qin, BJ; Xu, ZM; Ruan, JJ	Environmental-friendly recovery of non-metallic resources from waste printed circuit boards: A review	JOURNAL OF CLEANER PRODUCTION	2021	10.1016/j.jclepro.2020.123738
Swanson, ME; Franklin, JF; Beschta, RL; Crisafulli, CM; DellaSala, DA; Hutto, RL; Lindenmayer, DB; Swanson, FJ	The forgotten stage of forest succession: early-successional ecosystems on forest sites	FRONTIERS IN ECOLOGY AND THE ENVIRONMENT	2011	10.1890/090157
Orr, JA; Vinebrooke, RD; Jackson, MC; Kroeker, KJ; Kordas, RL;	Towards a unified study of multiple stressors: divisions and common	PROCEEDINGS OF THE ROYAL SOCIETY B-BIOLOGICAL SCIENCES	2020	10.1098/rspb.2020.0421

Mantyka-Pringle, C; Van den Brink, PJ; De Laender, F; Stoks, R; Holmstrup, M; Matthaei, CD; Monk, WA; Penk, MR; Leuzinger, S; Schafer, RB; Piggott, JJ	goals across research disciplines			
Fisher, RA; Koven, CD; Anderegg, WRL; Christoffersen, BO; Dietze, MC; Farrior, CE; Holm, JA; Hurtt, GC; Knox, RG; Lawrence, PJ; Lichstein, JW; Longo, M; Matheny, AM; Medvigy, D; Muller-Landau, HC; Powell, TL; Serbin, SP; Sato, H; Shuman, JK; Smith, B; Trugman, AT; Viskari, T; Verbeeck, H; Weng, ES; Xu, CG; Xu, XT; Zhang, T; Moorcroft, PR	Vegetation demographics in Earth System Models: A review of progress and priorities	GLOBAL CHANGE BIOLOGY	2018	10.1111/gcb.13910
Belzer, C; de Vos, WM	Microbes inside-from diversity to function: the	ISME JOURNAL	2012	10.1038/ismej.2012.6

	case of Akkermansia			
Bandeira, M; Giovanela, M; Roesch-Ely, M; Devine, DM; Crespo, JD	Green synthesis of zinc oxide nanoparticles: A review of the synthesis methodology and mechanism of formation	SUSTAINABLE CHEMISTRY AND PHARMACY	2020	10.1016/j.scp.2020.100223
Mesoudi, A; Thornton, A	What is cumulative cultural evolution?	PROCEEDINGS OF THE ROYAL SOCIETY B-BIOLOGICAL SCIENCES	2018	10.1098/rspb.2018.0712
Hardesty, BD; Harari, J; Isobe, A; Lebreton, L; Maximenko, N; Potemra, J; van Sebille, E; Vethaak, AD; Wilcox, C	Using Numerical Model Simulations to Improve the Understanding of Micro-plastic Distribution and Pathways in the Marine Environment	FRONTIERS IN MARINE SCIENCE	2017	10.3389/fmars.2017.00030
Wohl, E; Lane, SN; Wilcox, AC	The science and practice of river restoration	WATER RESOURCES RESEARCH	2015	10.1002/2014WR016874
Ahmad, M; Rajapaksha, AU; Lim, JE; Zhang, M; Bolan, N; Mohan, D; Vithanage, M; Lee, SS; Ok, YS	Biochar as a sorbent for contaminant management in soil and water: A review	CHEMOSPHERE	2014	10.1016/j.chemosphere.2013.10.071
Engler, RE	The Complex Interaction between Marine Debris and	ENVIRONMENTAL SCIENCE & TECHNOLOGY	2012	10.1021/es3027105

	Toxic Chemicals in the Ocean			
Kim, KH; Kabir, E; Jahan, SA	Exposure to pesticides and the associated human health effects	SCIENCE OF THE TOTAL ENVIRONMENT	2017	10.1016/j.scitotenv.2016.09.009
Prakash, V; Singh, VP; Tripathi, DK; Sharma, S; Corpas, FJ	Crosstalk between nitric oxide (NO) and abscisic acid (ABA) signalling molecules in higher plants	ENVIRONMENTAL AND EXPERIMENTAL BOTANY	2019	10.1016/j.envexpbot.2018.10.033
Baleta, J; Mikulcic, H; Klemes, JJ; Urbaniec, K; Duic, N	Integration of energy, water and environmental systems for a sustainable development	JOURNAL OF CLEANER PRODUCTION	2019	10.1016/j.jclepro.2019.01.035
Yu, XW; Manthiram, A	Electrode-electrolyte interfaces in lithium-based batteries	ENERGY & ENVIRONMENTAL SCIENCE	2018	10.1039/c7ee02555f
Giovannoni, SJ; Thrash, JC; Temperton, B	Implications of streamlining theory for microbial ecology	ISME JOURNAL	2014	10.1038/ismej.2014.60
Nayak, A; Bhushan, B	An overview of the recent trends on the waste valorization techniques for food wastes	JOURNAL OF ENVIRONMENTAL MANAGEMENT	2019	10.1016/j.jenvman.2018.12.041
Notarnicola, B; Sala, S; Anton, A; McLaren, SJ;	The role of life cycle assessment in supporting	JOURNAL OF CLEANER PRODUCTION	2017	10.1016/j.jclepro.2016.06.071

Saouter, E; Sonesson, U	sustainable agri-food systems: A review of the challenges			
Siddique, MNI; Ab Wahid, Z	Achievements and perspectives of anaerobic co-digestion: A review	JOURNAL OF CLEANER PRODUCTION	2018	10.1016/j.jclepro.2018.05.155
Kelly, JR; Scheibling, RE	Fatty acids as dietary tracers in benthic food webs	MARINE ECOLOGY PROGRESS SERIES	2012	10.3354/meps09559
Mahmood, A; Wang, JL	Machine learning for high performance organic solar cells: current scenario and future prospects	ENERGY & ENVIRONMENTAL SCIENCE	2021	10.1039/d0ee02838j
Asbjornsen, H; Goldsmith, GR; Alvarado-Barrientos, MS; Rebel, K; Van Osch, FP; Rietkerk, M; Chen, JQ; Gotsch, S; Tobon, C; Geissert, DR; Gomez-Tagle, A; Vache, K; Dawson, TE	Ecohydrological advances and applications in plant-water relations research: a review	JOURNAL OF PLANT ECOLOGY	2011	10.1093/jpe/rtr005
Campanale, C; Massarelli, C; Savino, I; Locaputo, V; Uricchio, VF	A Detailed Review Study on Potential Effects of Microplastics and Additives of	INTERNATIONAL JOURNAL OF ENVIRONMENTAL RESEARCH AND PUBLIC HEALTH	2020	10.3390/ijerph17041212

	Concern on Human Health			
Lai, CS; Locatelli, G; Pimm, A; Wu, XM; Lai, LL	A review on long-term electrical power system modeling with energy storage	JOURNAL OF CLEANER PRODUCTION	2021	10.1016/j.jclepro.2020.124298
Jiang, Y; Zevenbergen, C; Ma, YC	Urban pluvial flooding and stormwater management: A contemporary review of China's challenges and sponge cities strategy	ENVIRONMENTAL SCIENCE & POLICY	2018	10.1016/j.envsci.2017.11.016
Lead, JR; Batley, GE; Alvarez, PJJ; Croteau, MN; Handy, RD; McLaughlin, MJ; Judy, JD; Schirmer, K	Nanomaterials in the environment: Behavior, fate, bioavailability, and effectsAn updated review	ENVIRONMENTAL TOXICOLOGY AND CHEMISTRY	2018	10.1002/etc.4147
Martin, TG; Burgman, MA; Fidler, F; Kuhnert, PM; Low-Choy, S; Mcbride, M; Mengersen, K	Eliciting Expert Knowledge in Conservation Science	CONSERVATION BIOLOGY	2012	10.1111/j.1523-1739.2011.01806.x
Torralba, M; Fagerholm, N; Burgess, PJ; Moreno, G; Plieninger, T	Do European agroforestry systems enhance biodiversity and ecosystem	AGRICULTURE ECOSYSTEMS & ENVIRONMENT	2016	10.1016/j.agee.2016.06.002

	services? A meta-analysis			
Samways, MJ; Barton, PS; Birkhofer, K; Chichorro, F; Deacon, C; Fartmann, T; Fukushima, CS; Gaigher, R; Habel, JC; Hallmann, CA; Hill, MJ; Hochkirch, A; Kaila, L; Kwak, ML; Maes, D; Mammola, S; Noriega, JA; Orfinger, AB; Pedraza, F; Pryke, JS; Roque, FO; Settele, J; Simaika, JP; Stork, NE; Suhling, F; Vorster, C; Cardoso, P	Solutions for humanity on how to conserve insects	BIOLOGICAL CONSERVATION	2020	10.1016/j.biocon.2020.108427
Filbee-Dexter, K; Scheibling, RE	Sea urchin barrens as alternative stable states of collapsed kelp ecosystems	MARINE ECOLOGY PROGRESS SERIES	2014	10.3354/meps10573
Sifakis, S; Androutsopoulos, VP; Tsatsakis, AM; Spandidos, DA	Human exposure to endocrine disrupting chemicals: effects on the male and female reproductive systems	ENVIRONMENTAL TOXICOLOGY AND PHARMACOLOGY	2017	10.1016/j.etap.2017.02.024

Dong, LJ; Tong, XJ; Li, XB; Zhou, J; Wang, SF; Liu, B	Some developments and new insights of environmental problems and deep mining strategy for cleaner production in mines	JOURNAL OF CLEANER PRODUCTION	2019	10.1016/j.jclepro.2018.10.291
Ramanujam, J; Singh, UP	Copper indium gallium selenide based solar cells - a review	ENERGY & ENVIRONMENTAL SCIENCE	2017	10.1039/c7ee00826k
Manaia, CM; Rocha, J; Scaccia, N; Marano, R; Radu, E; Biancullo, F; Cerqueira, F; Fortunato, G; Iakovides, IC; Zammit, I; Kampouris, I; Vaz-Moreira, I; Nunes, OC	Antibiotic resistance in wastewater treatment plants: Tackling the black box	ENVIRONMENT INTERNATIONAL	2018	10.1016/j.envint.2018.03.044
Kumar, SG; Rao, KSRK	Physics and chemistry of CdTe/CdS thin film heterojunction photovoltaic devices: fundamental and critical aspects	ENERGY & ENVIRONMENTAL SCIENCE	2014	10.1039/c3ee41981a
Paul-Pont, I; Tallec, K; Gonzalez-Fernandez, C; Lambert, C; Vincent,	Constraints and Priorities for Conducting Experimental Exposures of	FRONTIERS IN MARINE SCIENCE	2018	10.3389/fmars.2018.00252

D; Mazurais, D; Zambonino-Infante, JL; Brotons, G; Lagarde, F; Fabioux, C; Soudant, P; Huvet, A	Marine Organisms to Microplastics			
Thomas, N; Dionysiou, DD; Pillai, SC	Heterogeneous Fenton catalysts: A review of recent advances	JOURNAL OF HAZARDOUS MATERIALS	2021	10.1016/j.jhazmat.2020.124082
Conti, C; Guarino, M; Bacenetti, J	Measurements techniques and models to assess odor annoyance: A review	ENVIRONMENT INTERNATIONAL	2020	10.1016/j.envint.2019.105261
Qin, YX; Li, GY; Gao, YP; Zhang, LZ; Ok, YS; An, TC	Persistent free radicals in carbon-based materials on transformation of refractory organic contaminants (ROCs) in water: A critical review	WATER RESEARCH	2018	10.1016/j.watres.2018.03.012
Wang, HX; Guerrero, A; Bou, A; Al-Mayouf, AM; Bisquert, J	Kinetic and material properties of interfaces governing slow response and long timescale phenomena in perovskite solar cells	ENERGY & ENVIRONMENTAL SCIENCE	2019	10.1039/c9ee00802k

Bucci, K; Tulio, M; Rochman, CM	What is known and unknown about the effects of plastic pollution: A meta-analysis and systematic review	ECOLOGICAL APPLICATIONS	2020	10.1002/eap.2044
Agrawal, AA	Current trends in the evolutionary ecology of plant defence	FUNCTIONAL ECOLOGY	2011	10.1111/j.1365-2435.2010.01796.x
Krzeminski, P; Tomei, MC; Karaolia, P; Langenhoff, A; Almeida, CMR; Felis, E; Gritten, F; Andersen, HR; Fernandes, T; Manaia, CM; Rizzo, L; Fatta-Kassinos, D	Performance of secondary wastewater treatment methods for the removal of contaminants of emerging concern implicated in crop uptake and antibiotic resistance spread: A review	SCIENCE OF THE TOTAL ENVIRONMENT	2019	10.1016/j.scitotenv.2018.08.130
Pearlman, J; Bushnell, M; Coppola, L; Karstensen, J; Buttigieg, PL; Pearlman, F; Simpsons, P; Barbier, M; Muller-Karger, FE; Munoz-Mas, C; Pissierssens, P; Chandler, C; Hermes, J; Heslop, E; Jenkyns, R;	Evolving and Sustaining Ocean Best Practices and Standards for the Next Decade	FRONTIERS IN MARINE SCIENCE	2019	10.3389/fmars.2019.00277

Achterberg, EP; Bensi, M; Bittig, HC; Blandin, J; Bosch, J; Bourles, B; Bozzano, R; Buck, JJH; Burger, EF; Cano, D; Cardin, V; Llorens, MC; Cianca, A; Chen, H; Cusack, C; Delory, E; Garello, R; Giovanetti, G; Harscoat, V; Hartman, S; Heitsenrether, R; Jirka, S; Lara-Lopez, A; Lanteri, N; Leadbetter, A; Manzella, G; Maso, J; McCurdy, A; Moussat, E; Ntoumas, M; Pensieri, S; Petihakis, G; Pinardi, N; Pouliquen, S; Przeslawski, R; Roden, NP; Silke, J; Tamburri, MN; Tang, HR; Tanhua, T; Telszewski, M; Testor, P; Thomas, J; Waldmann, C; Whoriskey, F				

Vereecken, H; Schnepf, A; Hopmans, JW; Javaux, M; Or, D; Roose, DOT; Vanderborght, J; Young, MH; Amelung, W; Aitkenhead, M; Allison, SD; Assouline, S; Baveye, P; Berli, M; Bruggemann, N; Finke, P; Flury, M; Gaiser, T; Govers, G; Ghezzehei, T; Hallett, P; Franssen, HJH; Heppell, J; Horn, R; Huisman, JA; Jacques, D; Jonard, F; Kollet, S; Lafolie, F; Lamorski, K; Leitner, D; McBratney, A; Minasny, B; Montzka, C; Nowak, W; Pachepsky, Y; Padarian, J; Romano, N; Roth, K; Rothfuss, Y; Rowe, EC; Schwen, A; Simunek, J; Tiktak, A; Van Dam, J; van der Zee,	Modeling Soil Processes: Review, Key Challenges, and New Perspectives	VADOSE ZONE JOURNAL	2016	10.2136/vzj2015.09.0131

SEATM; Vogel, HJ; Vrugt, JA; Wohling, T; Young, IM				
Bellwood, DR; Streit, RP; Brandl, SJ; Tebbett, SB	The meaning of the term 'function' in ecology: A coral reef perspective	FUNCTIONAL ECOLOGY	2019	10.1111/1365-2435.13265
Adao, T; Hruska, J; Padua, L; Bessa, J; Peres, E; Morais, R; Sousa, JJ	Hyperspectral Imaging: A Review on UAV-Based Sensors, Data Processing and Applications for Agriculture and Forestry	REMOTE SENSING	2017	10.3390/rs9111110
Keesstra, S; Nunes, JP; Saco, P; Parsons, T; Poeppl, R; Masselink, R; Cerda, A	The way forward: Can connectivity be useful to design better measuring and modelling schemes for water and sediment dynamics?	SCIENCE OF THE TOTAL ENVIRONMENT	2018	10.1016/j.scitotenv.2018.06.342
Heino, J	A macroecological perspective of diversity patterns in the freshwater realm	FRESHWATER BIOLOGY	2011	10.1111/j.1365-2427.2011.02610.x
Lenoir, J; Svenning, JC	Climate-related range shifts - a global multidimensional	ECOGRAPHY	2015	10.1111/ecog.00967

	synthesis and new research directions			
Groeneveld, J; Muller, B; Buchmann, CM; Dressler, G; Guo, C; Hase, N; Hoffmann, F; John, F; Klassert, C; Lauf, T; Liebelt, V; Nolzen, H; Pannicke, N; Schulze, J; Weise, H; Schwarz, N	Theoretical foundations of human decision-making in agent-based land use models - A review	ENVIRONMENTAL MODELLING & SOFTWARE	2017	10.1016/j.envsoft.2016.10.008
Guimaraes, N; Padua, L; Marques, P; Silva, N; Peres, E; Sousa, JJ	Forestry Remote Sensing from Unmanned Aerial Vehicles: A Review Focusing on the Data, Processing and Potentialities	REMOTE SENSING	2020	10.3390/rs12061046
Andersen, AN	Responses of ant communities to disturbance: Five principles for understanding the disturbance dynamics of a globally dominant faunal group	JOURNAL OF ANIMAL ECOLOGY	2019	10.1111/1365-2656.12907

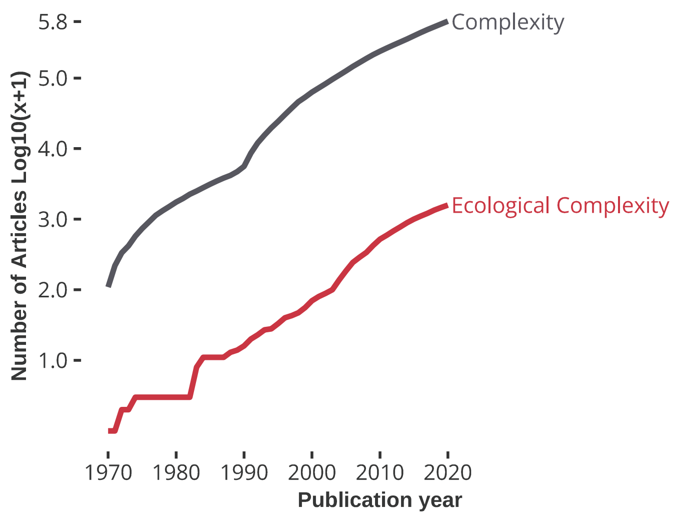

Fig. S1. Cumulative production of articles on "Complexity" and "Ecological complexity" over time. Cumulative production (from 1970 to 2021) between articles mentioning "complexity" in their titles and abstract including all scientific fields (gray line), and separately, ecology and environmental sciences, as approximated by the search term "ecological complexity" (red line). The number of articles were log-transformed [$\log_{10}(x+1)$] to ease the comparison between groups.

A

	Control					Ecological complexity				
	Q1	Q2	Q3	Q4	Total	Q1	Q2	Q3	Q4	Total
Adaptation	5	9	13	9	36	5	16	12	29	**62**
Aggregation	1	5	3	6	15	3	2	6	5	**16**
Attractor	0	0	0	0	0	1	2	4	1	**8**
Chaos	0	1	0	1	2	1	5	5	6	**17**
Dinamicity	8	13	22	22	65	2	8	27	50	**87**
Diversity	4	14	7	26	51	5	12	14	36	**67**
Emergence	4	10	8	6	28	5	15	21	21	**62**
Feedback	0	3	5	1	9	4	7	9	4	**24**
Flow	1	3	9	19	32	9	12	9	14	**44**
Fractality	0	0	0	0	0	1	0	3	3	**7**
Hierarchy	4	4	2	2	12	3	3	8	11	**25**
Homeostasis	0	0	0	0	0	1	2	0	0	**3**
Interaction	2	11	17	43	73	6	7	27	47	**87**
Memory	0	2	0	0	2	3	2	0	3	**8**
Modularity	1	1	3	2	7	3	3	5	0	**11**
Network	3	3	3	6	15	3	8	19	24	**54**
Non-equilibrium	0	0	0	0	0	1	3	1	1	**6**
Non-linearity	0	1	3	1	5	4	2	9	7	**22**
Resilience	0	2	1	1	4	5	8	4	11	**28**
Scaling	4	13	20	23	60	4	5	19	52	**80**
Self-organization	3	5	2	0	10	7	8	13	23	**51**
Stability	2	2	2	3	9	6	5	12	10	**33**
Threshold	0	2	8	4	14	1	3	9	4	**17**

B

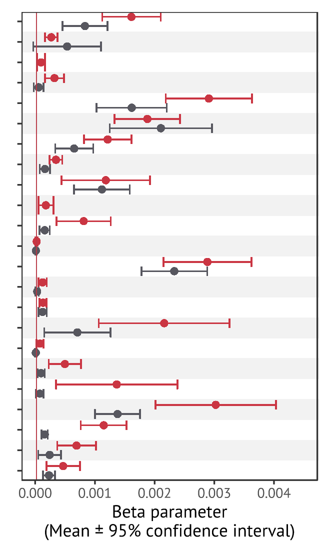

Fig. S2. Importance of features chosen to characterize *control* and *complexity* articles. These tables report (**A**) the number of times each feature appears in each quantile (Q1–4) considering the 1% most important terms in each article. The higher total value between groups is highlighted in bold (note that only the feature "Aggregation" appears more in the control group and some features do not appear at all in the *control* group). The rightmost graph (**B**) shows the distribution of beta parameters (that is, the per-topic-per-word probability for each word; see *Methods*) for each feature without sub-selecting the 1% most important terms. Vertical line represents the average values across all words, showing the relevance of these features for ecological complexity.

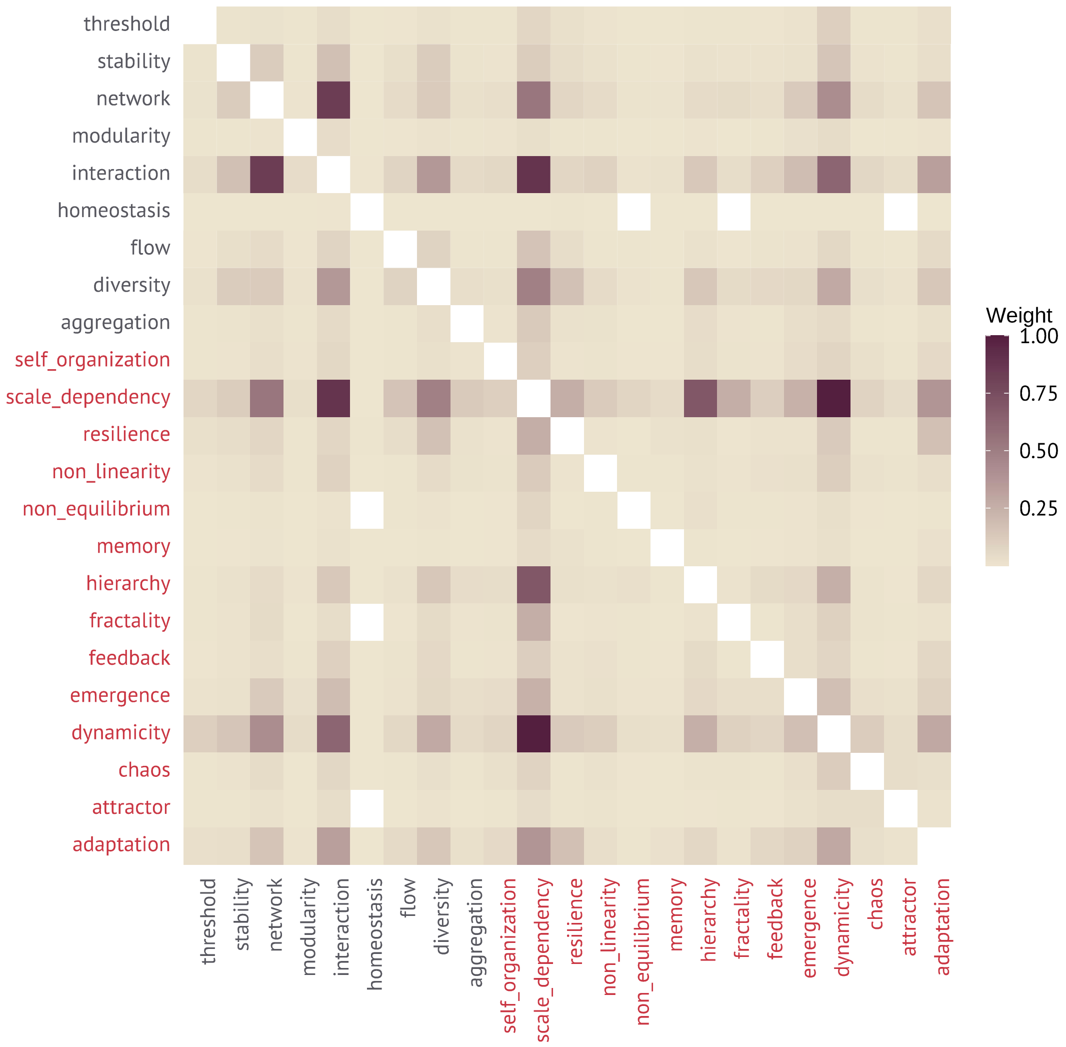

Fig. S3. Adjacency matrix for the co-occurrence of features. The colors in the name of the features indicate whether these are significantly related to *complexity* than the *control* articles based on Indicator Species Analysis, red features being significantly related to *complexity* articles. The filling gradient in the matrix represents the weight of the connections across features, estimated as the sum of the edge weights of the adjacent edges of the node.

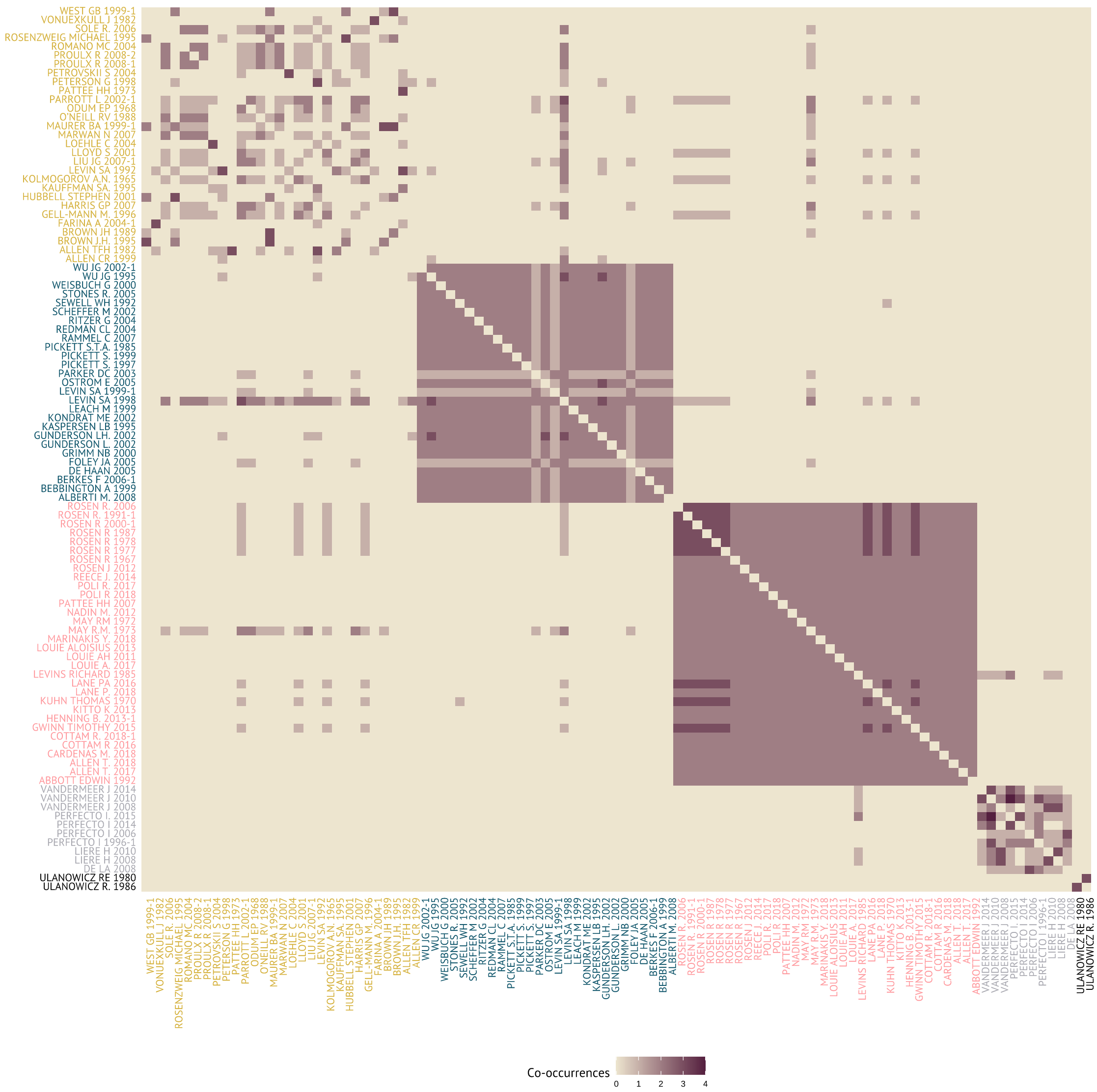

Fig. S4. Adjacency matrix for the co-citation of references. The colors in the name of the reference indicate the five clusters extracted using the Louvaine algorithm (see Fig. 5 in the main text). The filling gradient in the matrix represents the number of articles citing the pair of references simultaneously.

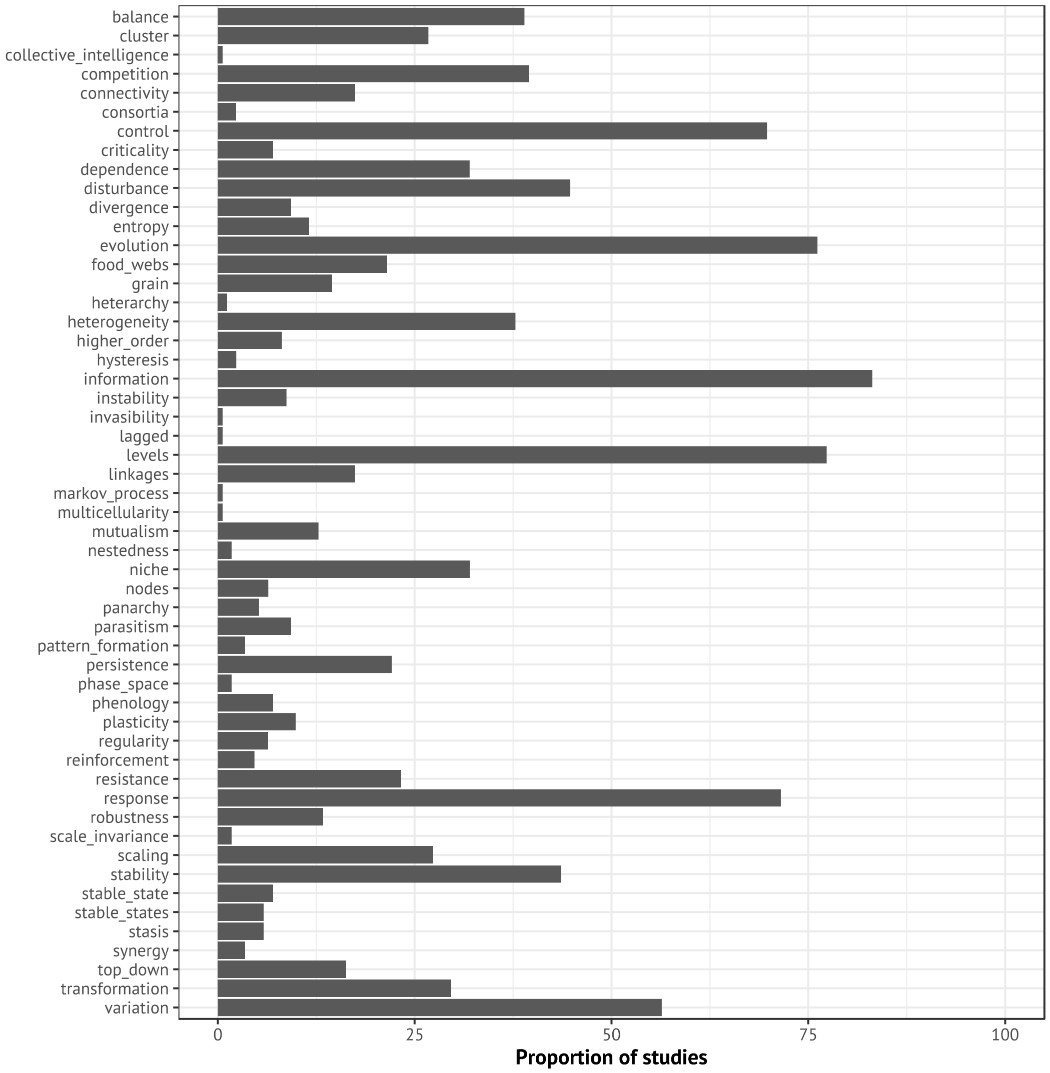

Fig. S5. The proportion of studies in our sample of *complexity* papers (*n* = 172) mentioning each of the words listed in "related concepts" (Table 1) to the features included in our study. Many important concepts for CSS, including criticality, panarchy, hierarchy, or entropy, were rarely mentioned in our sample of complexity papers (< 10% of articles); some, were never mentioned (e.g., brittleness); other, like "information" and "control", might refer to their CSS meaning, but are also common words, and we therefore preferred other words in defining our roster of 23 features typical of complex ecological systems.
